# Supramolecular Halogen Bonds in Asymmetric Catalysis

**DOI:** 10.3389/fchem.2020.599064

**Published:** 2020-10-21

**Authors:** Mikk Kaasik, Tõnis Kanger

**Affiliations:** Department of Chemistry and Biotechnology, Tallinn University of Technology, Tallinn, Estonia

**Keywords:** halogen bond, asymmetric catalysis, bifunctional catalyst, organocatalysis, metal-catalyzed reactions

## Abstract

Halogen bonding has received a significant increase in attention in the past 20 years. An important part of this interest has centered on catalytic applications of halogen bonding. Halogen bond (XB) catalysis is still a developing field in organocatalysis, although XB catalysis has outgrown its proof of concept phase. The start of this year witnessed the publication of the first example of a purely XB-based enantioselective catalytic reaction. While the selectivity can be improved upon, there are already plenty of examples in which halogen bonds, among other interactions, play a crucial role in the outcome of highly enantioselective reactions. This paper will give an overview of the current state of the use of XBs in catalytic stereoselective processes.

## Introduction

Halogen bonding is an attractive interaction between an electrophilic region associated with a halogen atom and a nucleophilic region (Desiraju et al., 2013). While the exact origin of this interaction is still under debate, the distribution of electron density on the halogen atom is anisotropic, leading to the formation of an electropositive region, often referred to by the term “σ-hole” (Brinck et al., 1992). Although the first evidence of this phenomenon dates to the early nineteenth century (Colin and de Claubry, 1814), halogen bonding has gained considerable attention only in the last two decades (for selected reviews, see: Gilday et al., 2015; Cavallo et al., 2016; Wang et al., 2016; Montaña, 2017). While solid state applications have been predominant (for selected reviews, see: Troff et al., 2013; Li et al., 2016; Christopherson et al., 2018), halogen bonding has gained wider use in the solution phase as well (for selected reviews, see: Beale et al., 2013; Jentzsch, 2015; Tepper and Schubert, 2018).

Halogen bonds (XBs) are often compared to hydrogen bonds (HBs), with XB strengths ranging from 5 to 180 kJ/mol (Metrangolo et al., 2005). However, XBs have several beneficial characteristics which contribute to the growing interest in XB applications. An XB is formed on the elongation of the covalent bond to the halogen and, therefore, XBs are very linear, with angles near 180° (Metrangolo et al., 2005; Rissanen, 2008). Compared to hydrogen bonding, a wider range of donor atoms can be used from the seventh row, although in reality chlorine, bromine and iodine are typically used (Cavallo et al., 2016). The halogen atoms can be ranked by their XB donor ability in the order of F < Cl < Br < I, which corresponds to the increase in the polarizability of the halogen atoms (Clark et al., 2007). Also, the substituent attached covalently to the halogen atom can be varied to tune the XB donor ability (Nepal and Scheiner, 2015). Based on the “hard and soft (Lewis) acids and bases” theory (Pearson, 1963), hydrogen is a hard atom, while halogens are softer atoms. Therefore, XBs and HBs should have different preferences toward acceptor atoms (Robertson et al., 2014). In addition, it has been demonstrated that XBs are not as sensitive to solvent effects as HBs are Robertson et al. (2014, 2017).

The deliberate use of XBs for a catalytic purpose was realized by Bruckmann et al. (2008). 1-Iodoperfluorooctane was used to activate quinolines in their reduction with a Hantzsch ester. This opened the door for the development of a new field in organocatalysis and since then a growing number of publications have dealt with catalytic applications of XBs (for recent reviews, see: Bamberger et al., 2019; Sutar and Huber, 2019; Breugst and Koenig, 2020; Yang and Wong, 2020). Thus, far, considerable effort has gone into proving the catalytic viability of XBs and as a result, a set of benchmark reactions (Michael, aza-Diels Alder, and the halogen abstraction reaction) for XB catalysis has emerged. Since neutral XB acceptors are generally quite weak, the design and use of charge-assisted XB catalysts based on azolium-cores is very dominant in studies. Asymmetric XB catalysis has been an exciting prospect for some time (Kniep et al., 2013). However, considerable advancements in the field have been made only in the past 2 years.

**Figure 1:**
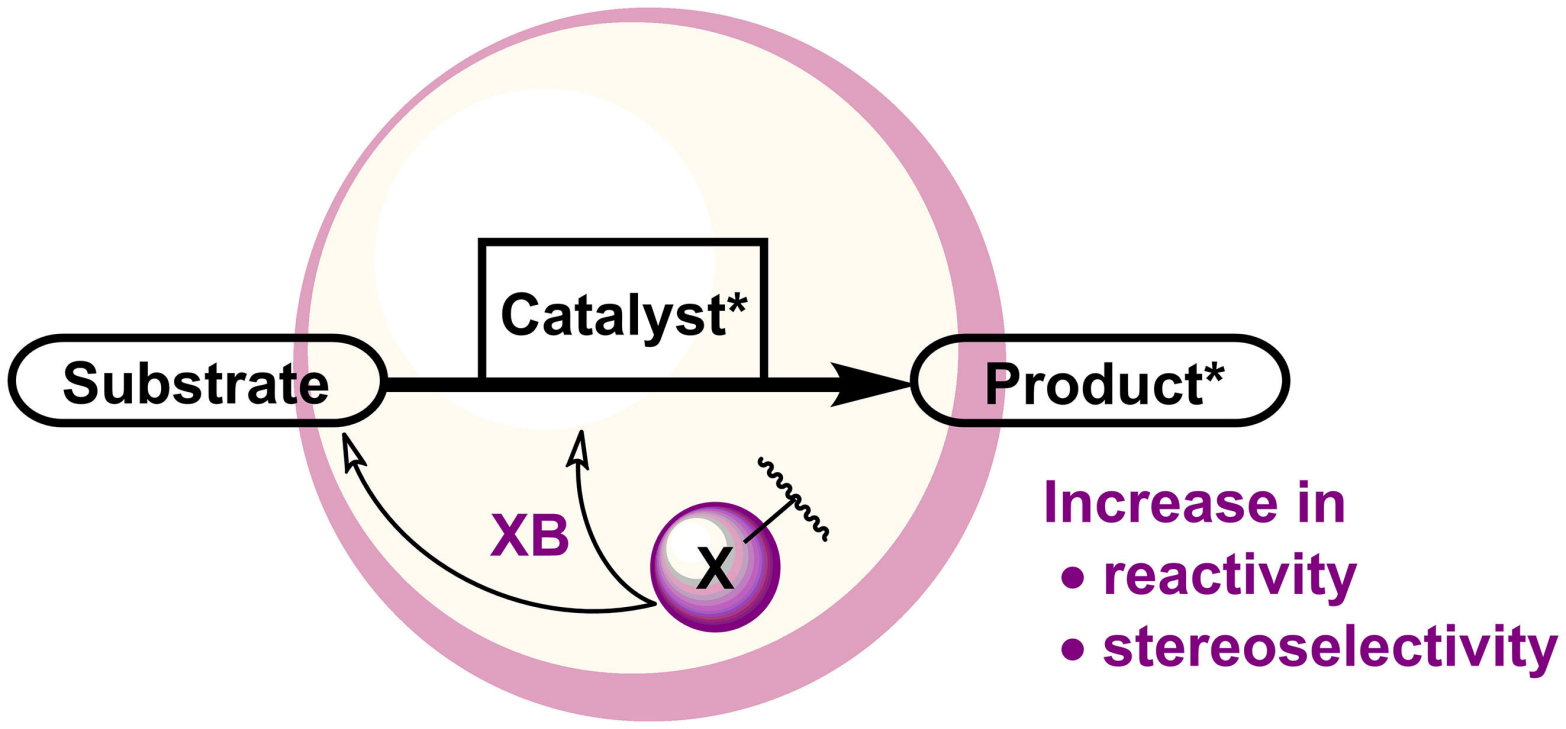
XBs in influencing enantioselective catalytic reactions.

This review will explore the use of XBs in catalytic enantioselective transformations in which XBs have been demonstrated to influence the outcome of the reaction (Figure 1). With a few exceptions, examples of transient halogen bonding (an XB to a Lewis base leads to the cleavage of the covalent bond with the halogen atom) are omitted from the review, as in these instances the XB donors are usually used as reagents. In addition, by focusing the discussion on examples in which the inert Lewis acid nature of the XB donor is utilized, the discussion will also better reflect the most prominent trends in XB catalysis.

**Figure 2:**
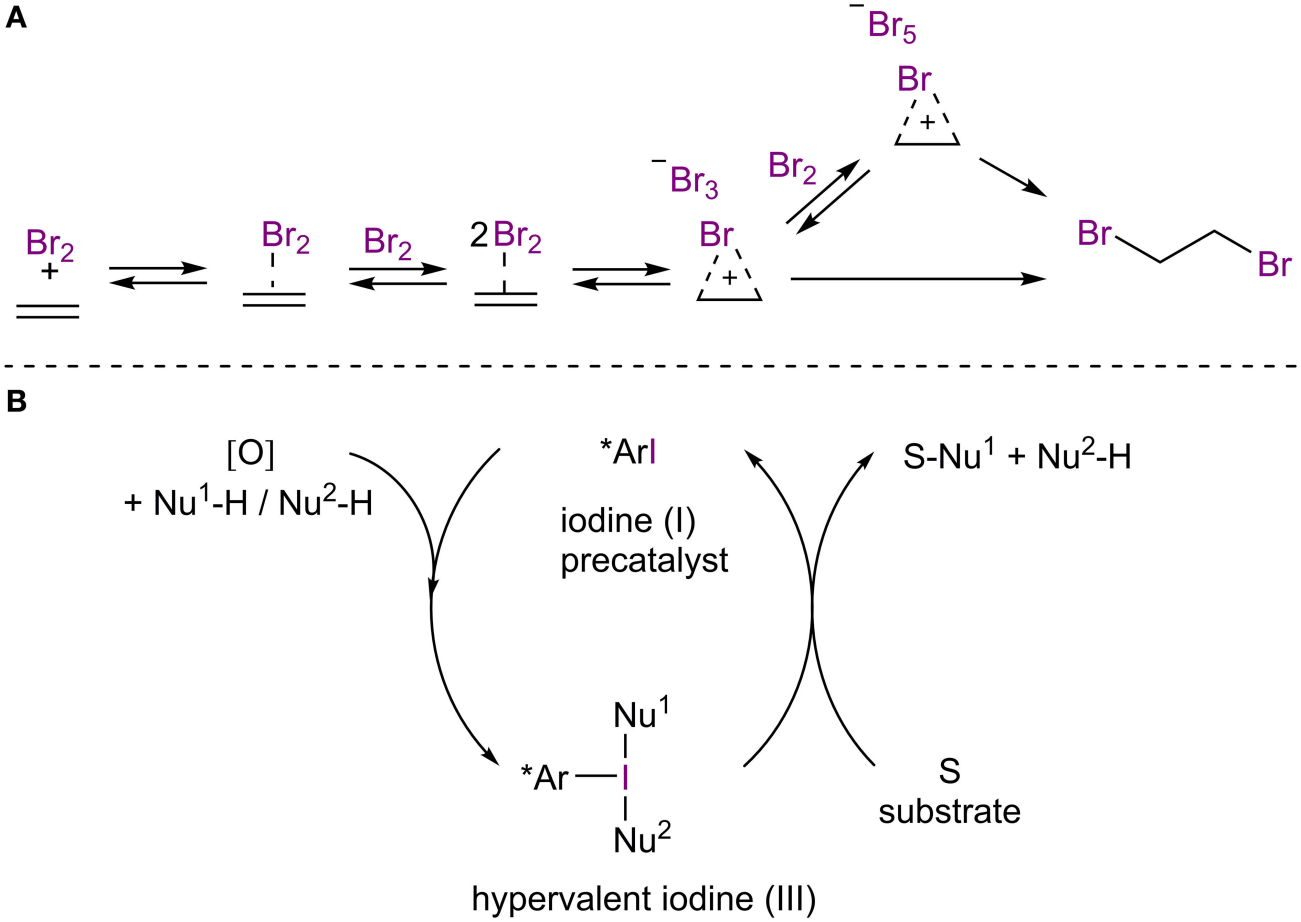
**(A)** Participation of XBs in the bromination of alkene; **(B)** general scheme of iodine(I/III)-enabled oxidative transformations.

Halogen-bonded adducts have been demonstrated to be on the reaction pathways of halogenation reactions of aromatic and other unsaturated systems (Brown, 1997; Lenoir and Chiappe, 2003; Figure 2A). Over the years, several asymmetric variants of these reactions (for example: halogenations, halocyclizations) have been developed (for selected reviews, see: Murai and Fujioka, 2013; Cheng et al., 2014; Cresswell et al., 2015; Cai et al., 2019; Kristianslund et al., 2019). However, the term halogen bonding is only rarely used in association with these processes. The transient nature of these XBs also place the reactions out of the focus of this review. As the field of asymmetric halogenation reactions is well-established, the reader is referred to previous reviews. Nonetheless, some papers have addressed the issue of halogen bonding in enantioselective halogenation reactions in more detail, and three particular cases are included in the later part of this review.

Chiral hypervalent organoiodine reagents have been used in different oxidative transformations in which the reagent is temporally inserted into a suitable substrate and is afterwards displaced by a nucleophilic species (Figure 2B; for recent reviews, see: Claraz and Masson, 2018; Flores et al., 2019; Parra, 2019). Typically, these reagents are generated *in situ* from chiral iodoarenes by sacrificial oxidants, which are also used to regenerate the hypervalent organoiodine. Thus, only catalytic quantities of iodoarenes are needed. As the coordination patterns in hypervalent iodine species can be viewed as special cases of halogen bonding (Heinen et al., 2020; Turunen and Erdélyi, 2020) and as XBs can contribute to halogen insertions, it is pertinent to mention this type of catalysis in the review. Nevertheless, the contribution of XBs to these catalytic processes are again rarely acknowledged and the topic has been thoroughly reviewed in recent years by experts in the field. Therefore, the reader is referred to the previously mentioned reviews.

For the following discussion, the review will be divided into three parts, based on how the XBs in the catalytic system affect the outcome of the enantioselective reaction by enhancing:

- reactivity
- both reactivity and stereoselectivity
- stereoselectivity.

A valid argument can be made that reactivity and stereoselectivity are connected to each other and therefore should not be separated from one another. However, the examples encompass very different types of reactions, activation modes, roles of the halogen etc., to make a division based on a more traditional reasoning. The division of the review is based on the observable outcomes of the reactions.

## XBs in Enhancing Reactivity

**Figure 3:**
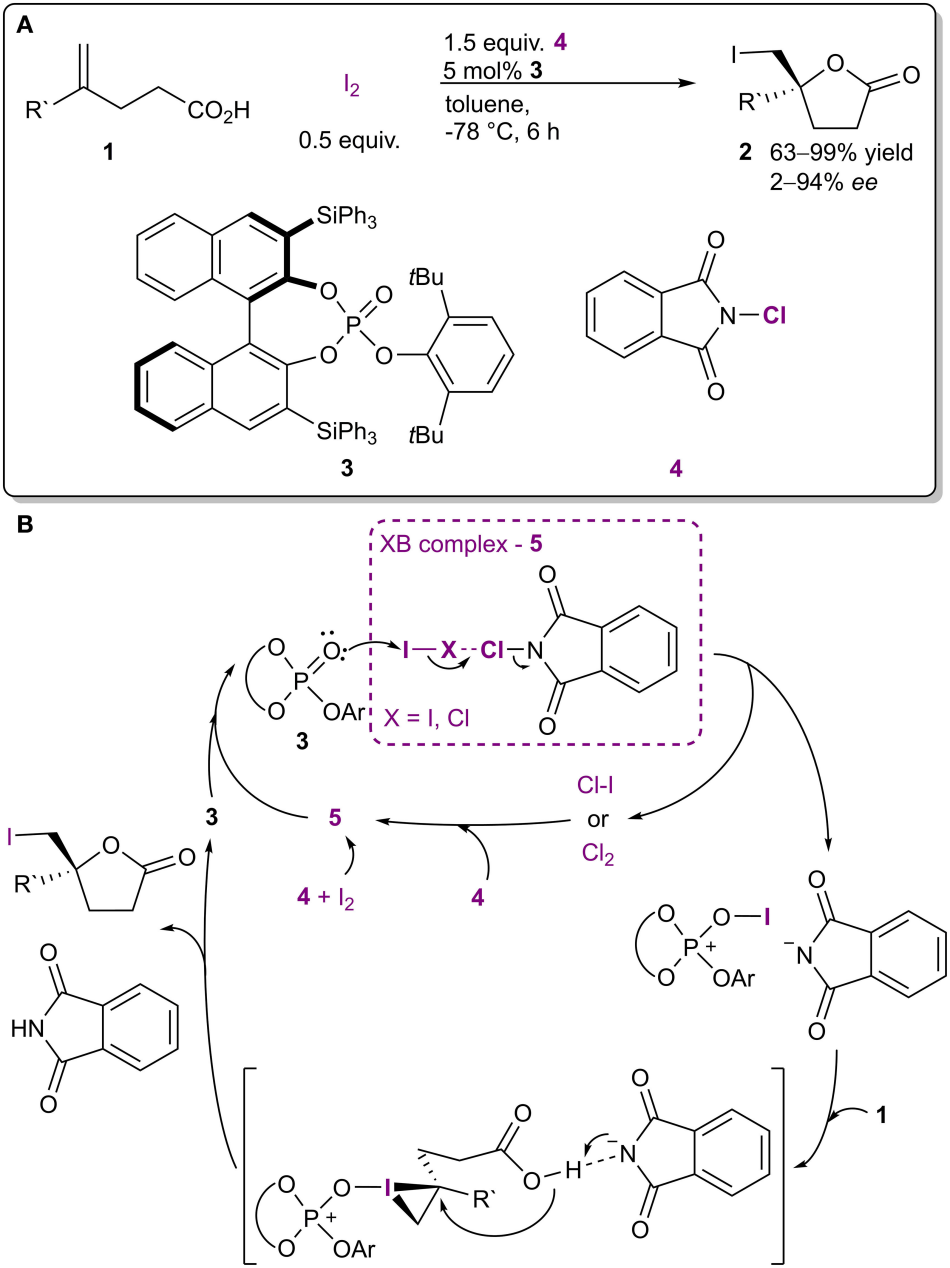
**(A)** Lewis base-catalyzed halocyclization utilizing the complex **5** as a halogenating agent; **(B)** proposed mechanism of the reaction.

Through the years several reagents have been used in halogenation reactions. Interestingly, it has been noted that combinations of halogenating agents give better results than the use of a single reagent. The increase in reactivity can be reasoned to come from XBs. For instance, molecular iodine (or ICl) and haloimides (such as **4**, Figure 3A; Nakatsuji et al., 2014) formed XB-based complexes **5** in the iodolactonization of 4-arylmethyl-4-pentenoic acids. The formation of complex **5** was postulated based on the appearance of new bands in the Raman spectrum of the mixture of I_2_ and **4** compared to the spectra of individual components. In addition, the lactonization was accelerated when I_2_ and **4** were premixed for 1 h prior to the addition of catalyst **3** and acid **1**, which also supported the formation of a complex. The haloimide **4** and iodine were consumed in the course of the reaction, and therefore both were actually reagents. However, the generation of ICl made it possible to use catalytic amounts of I_2_ in the reaction. According to the proposed mechanism, complex **5** reacted with the chiral triaryl phosphate **3**, which led to the formation of an active iodinating species (Figure 3B). This in turn reacted with the double bond of **1** resulting in a chiral iodonium ion due to the presence of **3**. Intramolecular ring closure by the carboxyl group gave product **2**. The halogenating complex **5** is much more reactive than I_2_ or **4** alone and made it possible to conduct the reaction at −78°C, which resulted in higher selectivities. Under optimized conditions, γ-lactones **2** were obtained in moderate to excellent yields (63-99%), with up to excellent enantioselectivities (2–99% *ee*). Similar halogenating complexes have been successfully used in other applications as well. However, often the XB contribution in these systems is not discussed (Kwon et al., 2008; Veitch and Jacobsen, 2010; Tungen et al., 2012; Arai et al., 2013, 2015a,b), or is only mentioned in passing, without in-depth studies resulting in new knowledge on the XB contribution (Mizar et al., 2014; Lu et al., 2018).

**Figure 4:**
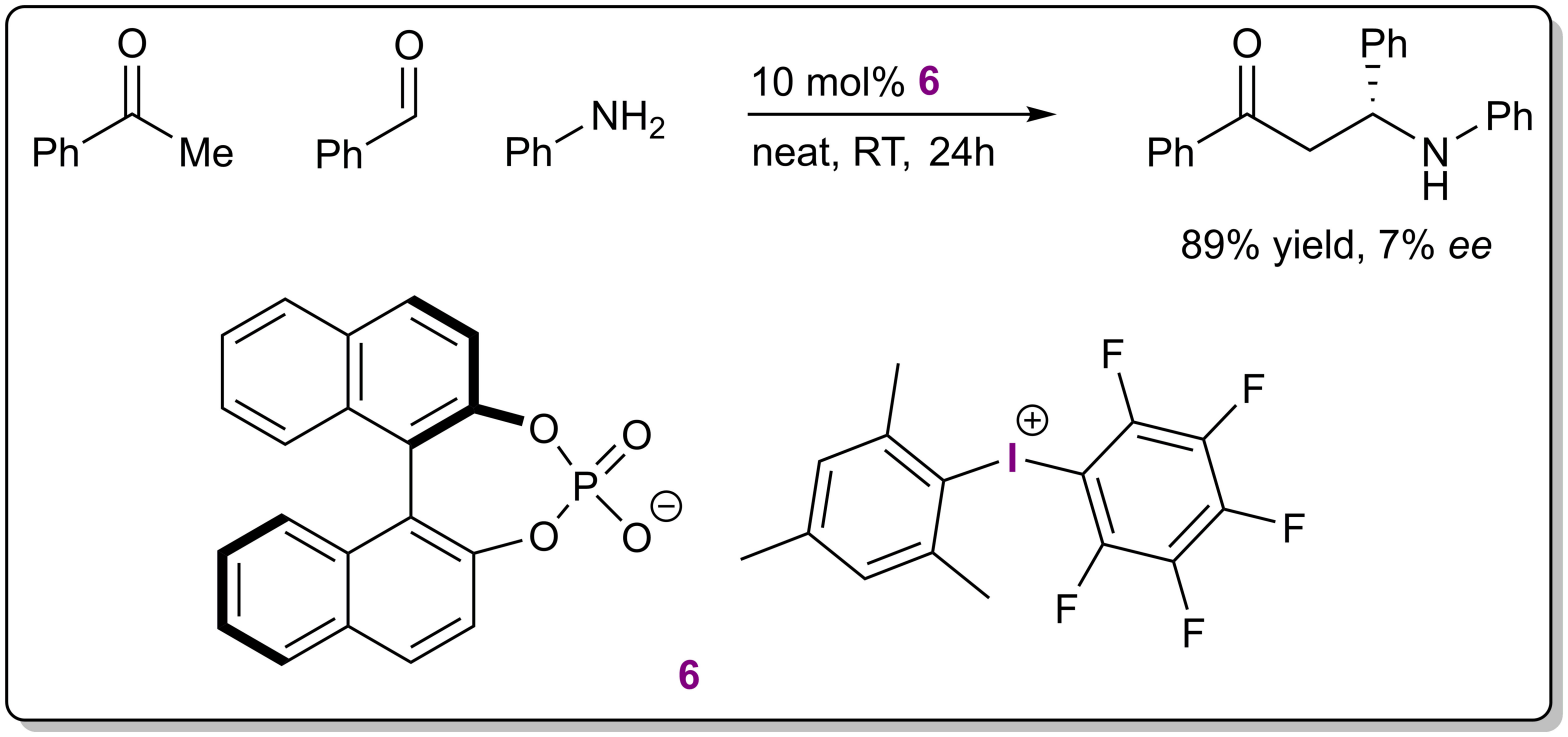
Three-component Mannich reaction catalyzed by diaryliodonium salt **6**.

The definition of halogen bonding is inclusive of hypervalent halogens, although monovalent halogens have found wider use in catalytic applications. As one of the first examples, diaryliodonium salts were used as catalysts in a three-component Mannich reaction (Figure 4; Zhang et al., 2015). A very low level of enantioinduction was achieved with the use of salt **6** containing a chiral BINOL-based phosphate as the counterion. The Lewis acidic diaryliodonium cation was assumed to activate either the carbonyl or the imine group of the substrates through lowering their LUMO. Thus, the counterion was responsible for enantioinduction and this example is representative of asymmetric counterion-directed catalysis (ACDC; Mahlau and List, 2013). Although the authors did not mention the term XB catalysis and presented the work as an example of Lewis acid catalysis, then due to the nature of XBs it is pertinent to include the example in this review. Catalytic activity of the salts was inferred from a few comparative experiments. For example, no reaction took place without the diaryliodonium salts and Brønsted acids were less efficient than the achiral salts in facilitating the reaction and provided comparable yields of product if at least one equivalent of the acid was used. Unfortunately, the mechanism of the reaction and the exact cause of enantioinduction were not elaborated, leaving unclear the true nature of the catalysis.

**Figure 5:**
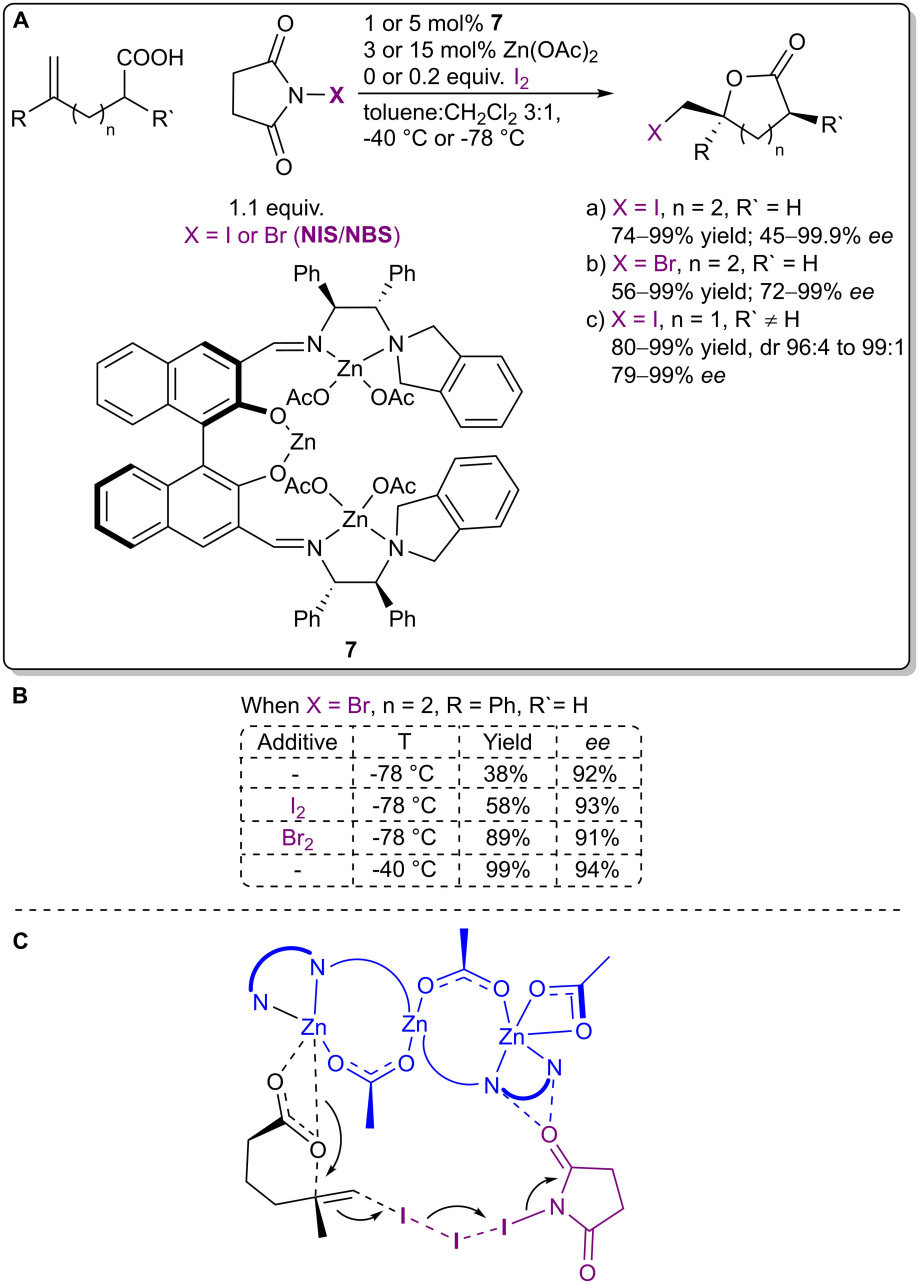
**(A)** Zinc acetate-catalyzed asymmetric halolactonization using the **I**_**2**_**-NIS/NBS** complex as a halogenating agent; **(B)** the influence of additives and temperature on the bromolactonization; **(C)** the proposed transition state for the iodolactonization.

Arai et al. reasoned the importance of the formation of an XB network in the metal acetate-catalyzed asymmetric halolactonization reaction (Figure 5A; Arai et al., 2019) with DFT calculations. The use of I_2_ (or Br_2_) as an additive helped to facilitate the formation of an XB network with NIS/NBS, which resulted in the acceleration of the reaction, without affecting the stereochemical control (Figure 5B). In the case of bromolactonization a similar effect could be obtained with an increase in reaction temperature from −78 to −40°C. DFT calculations of the transition state (TS) of the iodolactonization revealed that alkene activation and iodine transfer occurred from NIS in the absence of I_2_ and from I_2_ when it was present (Figure 5C). The addition of I_2_ did not significantly influence the organization of the TS, but in the presence of the XB network the TS was greatly stabilized (by 12.6 kcal/mol), leading to a faster reaction. As I_2_ was regenerated in the process, it could be used in catalytic quantities. Under optimized conditions, δ-lactones were obtained in moderate to excellent yields (I: 74–99%, Br: 56–99%) with moderate to excellent enantioselectivities (I: 45–99% *ee*, Br: 72–99% *ee*), and γ-lactones were obtained in good to excellent yields (I: 80–99%), with excellent diastereoselectivities (I: dr 96:4–99:1) and good to excellent enantioselectivities (I: 79–99% *ee*).

**Figure 6:**
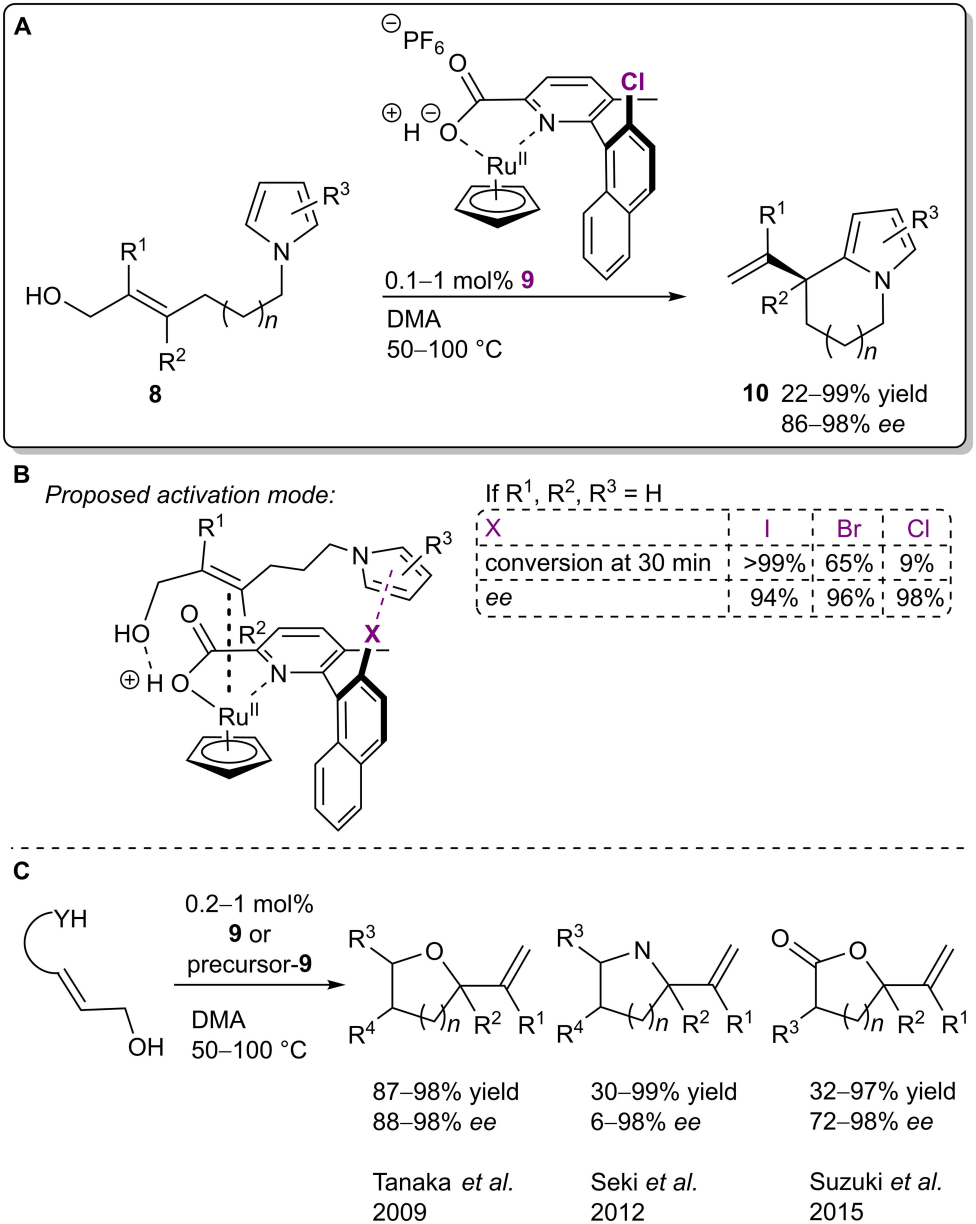
**(A)** Ruthenium-catalyzed dehydrative cyclization of allylic alcohols **8**; **(B)** proposed activation mode and influence of the halogen atom on the reaction; **(C)** other dehydrative cyclizations performed in the presence of **9** or its precursors.

Recently, XBs were utilized in an enantioselective dehydrative cyclization of allylic alcohols **8** *N*-tethered to pyrroles (Figure 6A; Iwase et al., 2020). The authors proposed a mechanism in which the allylic alcohol **8** was activated by both the Brønsted acid fragment and the ruthenium species of the catalyst **9** (Figure 6B). At the same time, the XB donor fragment of the catalyst stabilized the substrate/catalyst complex through a σ-π XB to a pyrrole core and also brought the nucleophilic and electrophilic parts of the substrate closer together. These effects in turn led to an S_N_2'-type cyclization. Interestingly, the choice of the XB donor atom had almost no influence on the selectivity of the reaction (94%/96%/98% *ee* respectively for I/Br/Cl). On the other hand, the activity was greatly affected by this choice and aligned with the XB donor ability of the halogens (99%/65%/9% conversion after 30 min for I/Br/Cl, respectively). This trend was used to support the XB-involved mechanism. The scope of the reaction revealed that in almost all of the cases the cyclic products **10** were obtained with high yields (up to 99%) and excellent selectivities (up to 98% *ee*). Interestingly, the catalytic system and its analogs were first developed ten years ago by Kitamura et al. and have been used in similar asymmetric intramolecular dehydrative allylations (Figure 6C; Tanaka et al., 2009; Seki et al., 2012; Suzuki et al., 2015). In the initial publication, the chloro group seemed to play a more crucial role than just a steric control unit and it was speculated that the chloro group helped to stabilize the TS by lowering the LUMO level (Tanaka et al., 2009). Nevertheless, the possibility of XB participation in these cyclizations was only proposed in their most recent study. Another implication of the series of publications is the fact that XBs might also be operable in other catalytic systems that could have gone unnoticed since information about XBs has reached a wider audience only in the past 20 years.

## XBs in Enhancing Both Reactivity and Stereoselectivity

**Figure 7:**
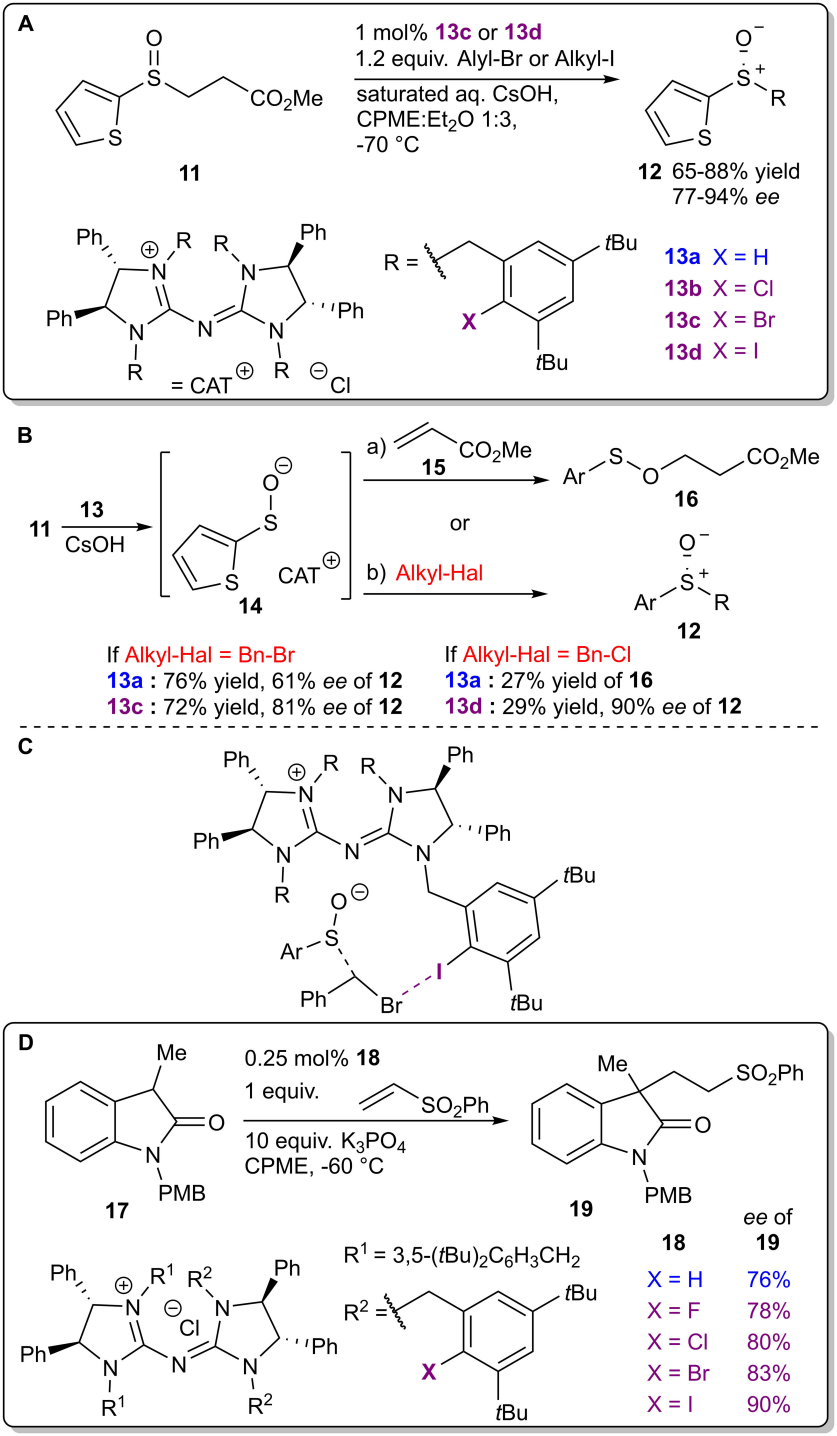
**(A)** Enantioselective alkylation of sulfenate anions; **(B)** alternative reaction pathways and influence of halogen atoms on the reaction outcome; **(C)** proposed XB participation in the TS leading to product **12**; **(D)** chiral pentanidium salt-catalyzed enantioselective Michael reaction.

Chiral pentanidium salts **13** containing an XB donor fragment were used as phase-transfer catalysts (PTCs) in the enantioselective alkylation of sulfenate anions (Figure 7A; Zong et al., 2014). First, cesium hydroxide converted the β-sulfinyl methyl ester **11** into the corresponding enolate, which decomposed into methyl acrylate **15** and a sulfenate anion that formed a chiral complex **14** with the cationic part of the PTC (Figure 7B). Molecular mechanics calculations revealed that a halogen bond (Figure 7C) along with hydrogen bonds were formed between the catalyst and the leaving halide group of the alkylating agent in the TS, resulting in the enantioselective formation of the sulfoxide **12**. The presence of this XB was used to rationalize some of the observed trends. Initial optimization reactions revealed that if the leaving group was a bromide anion, the presence of a halogen atom in the catalyst had a more profound influence on the stereoselectivity than on the reactivity (76% yield and 61% *ee* compared to 72% yield and 81% *ee* if the non-halogenated or the brominated analog was used, respectively). On the other hand, when the leaving group was a chloride anion, the presence of a halogen atom in catalyst **13** had a more profound influence on the reactivity than on the selectivity (27% of only side product **16** obtained after 48 h compared to 29% yield of product **12** with 90% *ee* after 24 h if the non-halogenated or the iodinated analog was used, respectively). The formation of the side product **16** took place when the anionic intermediate attacked the initially released methyl acrylate **15** if the alkyl halide was omitted from the reaction mixture or when a less active electrophile was used along with the non-halogenated or chlorinated version of the PTC **13**. It was also demonstrated that, although the iodinated catalyst gave the best results, in specific cases the brominated analog should be used instead. With the optimized conditions, a set of products **12** were obtained in moderate to high yields (65–88%) and high to excellent stereoselectivities (77–94% *ee*). In 2016 Tan et al. used the same catalyst family to achieve enantioselective alkylation of dihydrocoumarins (Teng et al., 2016). Major differences in the selectivity and the reactivity of the reaction using the halogenated catalysts were not observed, but all of the halogenated catalysts showed higher selectivities compared to the corresponding hydrogenated and methylated analogs. In 2015 Tan et al. used similar catalysts **18**, in which only some of the R substituents on the nitrogen atoms of the pentanidium contained halogen atoms, to achieve an enantioselective conjugate addition between oxindoles **17** and vinyl sulfones (Figure 7D; Zong et al., 2015). A slight increase in the selectivity of the reaction was observed with the variation of halogen atoms corresponding to the size/polarizability order (as F<Cl<Br<I with 78%<80%<83%<90% *ee* of **19**). Unfortunately, neither of the studies explored the role of the halogen atom or the participation of XBs on the outcome of the reaction.

**Figure 8:**
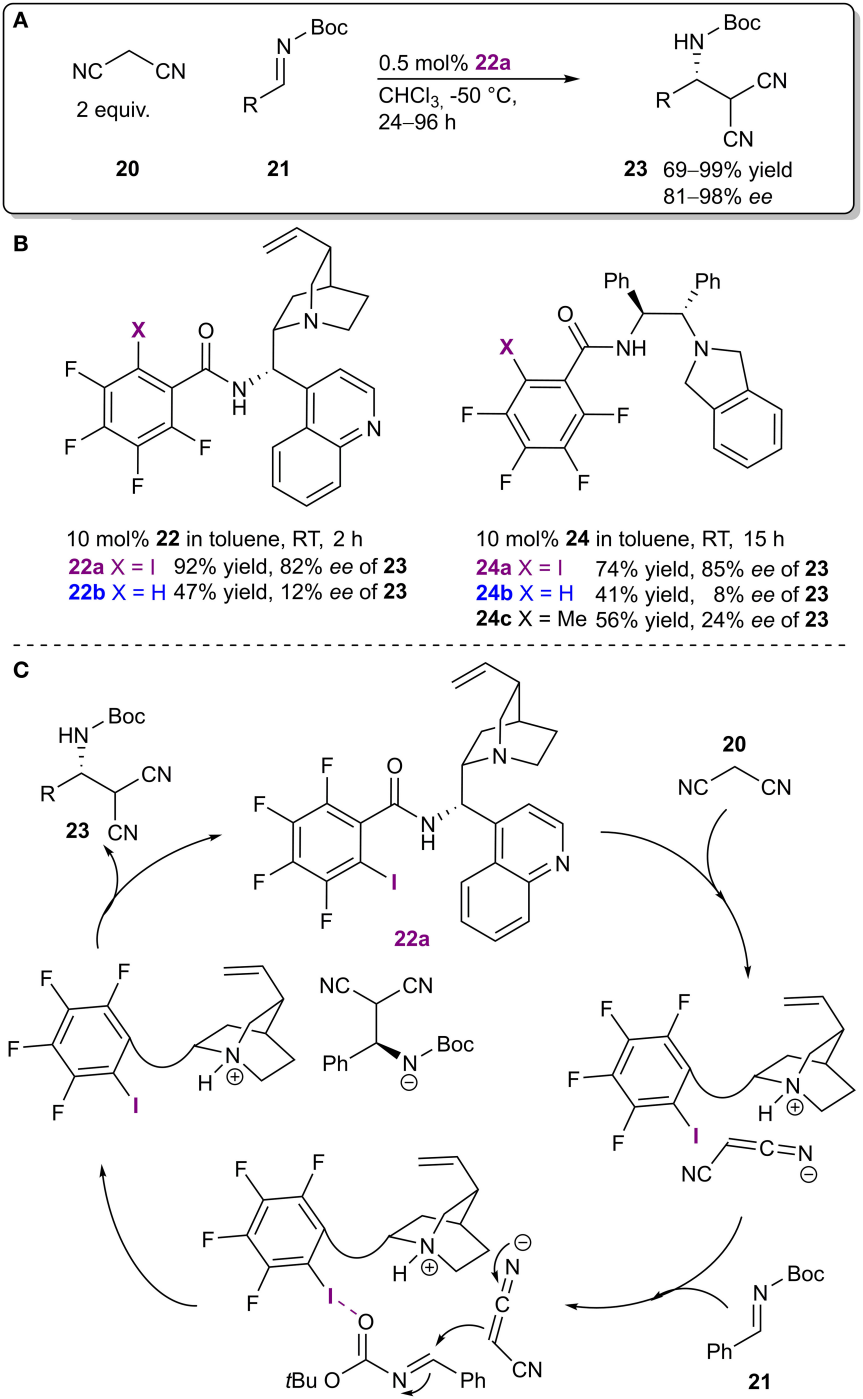
**(A)** Enantioselective Mannich reaction; **(B)** catalysts probed to determine the influence of the halogen atom on the reaction; **(C)** proposed catalytic cycle.

The Arai research group combined a Brønsted base fragment and an XB donor motif into a bifunctional organocatalyst **22**, which was used in an enantioselective Mannich reaction (Figure 8A; Kuwano et al., 2018). This example is also important for the fact that from the outset of the study, the XB donating core was a key design element of an asymmetric catalyst which was successfully used to achieve high levels of enantioinduction. Different catalyst core structures were explored and in the case of two such cores (**22** and **24**), a comparison between the non-halogenated and the halogenated systems was made. When non-halogenated analogs of the catalysts were used containing either an isosteric methyl group or a hydrogen atom, both the activity and the selectivity dropped significantly (from 92%/74% yield and 82%/85% *ee* for the iodinated catalysts **22a**/**24a** to 47%/41% yield and 12%/8% *ee* for the hydrogen analogs **22b**/**24b** and -/56% yield and -/24% *ee* for the methylated analog **24c**; Figure 8B). NMR experiments were also conducted with truncated analogs of **22**, containing only a part of the catalyst, to determine the most plausible interaction sites in catalyst **22a** and the substrates. Shift changes observed in the NMR spectra indicated the formation of an XB between imine **21** and the XB donor motif of **22a**. Based on the observations, a likely mechanism was proposed in which the base was used to deprotonate and activate the nucleophilic malononitrile **20** and the XB donor motif was used to activate and coordinate the imine **21** through its carbonyl group (Figure 8C). Several substituents on the imine **21** were tolerated, which led to products **23** with excellent yields and stereoselectivities (up to 99% yield and 98% *ee*). It was also demonstrated that the new bifunctional XB catalyst **22a** was superior to a known chiral thiourea catalyst acting mainly through HBs.

**Figure 9:**
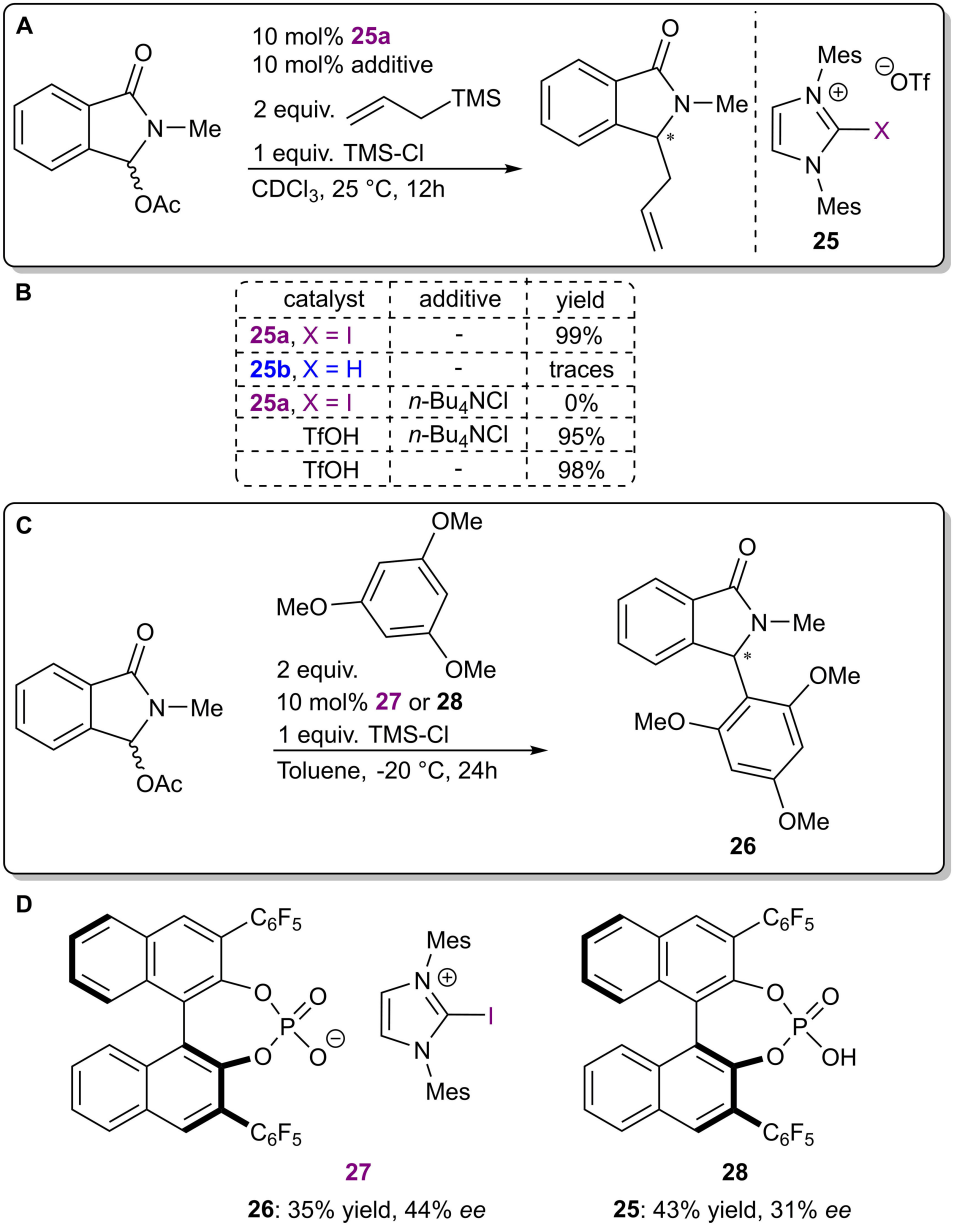
**(A)** Iodo-imidazolium triflate-catalyzed nucleophilic addition to α-oxo-lactams; **(B)** control experiments used to determine the influence of the halogen atom; **(C)** reaction conditions used in the asymmetric variant; **(D)** catalytic systems used in the asymmetric reaction.

The next two examples are again representative of asymmetric counterion-directed catalysis. Iodo-imidazolium salts **25a** were used to activate *N*-acyliminium ions in a halogen abstraction type reaction (Figure 9A; Chan and Yeung, 2019). It was postulated that the catalyst transformed an initially formed *N*-acyliminium chloride into a more reactive triflate intermediate by binding the chloride ions through XBs. In a preliminary experiment (Figure 9C), a low level of asymmetric induction was achieved with the use of salt **27** containing a chiral BINOL-based phosphate as the counterion. It should be noted that the corresponding phosphoric acid **28** also demonstrated catalytic activity with a somewhat higher level of activity and a lower level of selectivity (Figure 9D). Therefore, XBs seem to have influenced both the reactivity and selectivity of the reaction, although in theory the latter should have only been affected by the chiral counterion. The exact nature of the asymmetric induction was not further explored, although control experiments carried out with an achiral analog of the catalyst **27** supported an activation mode by halogen bonding in general (Figure 9B). First, the hydrogen analog **25b** was inactive in the reaction. Next, ^13^C NMR experiments revealed a significant interaction between catalyst **25a** and chloride anions and demonstrated that **25a** did not substantially interact with TMS-Cl, ruling out the activation of it. Finally, the reaction could be shut down with the use of excess chloride anions, which was not possible when TfOH was used to catalyze the reaction.

**Figure 10:**
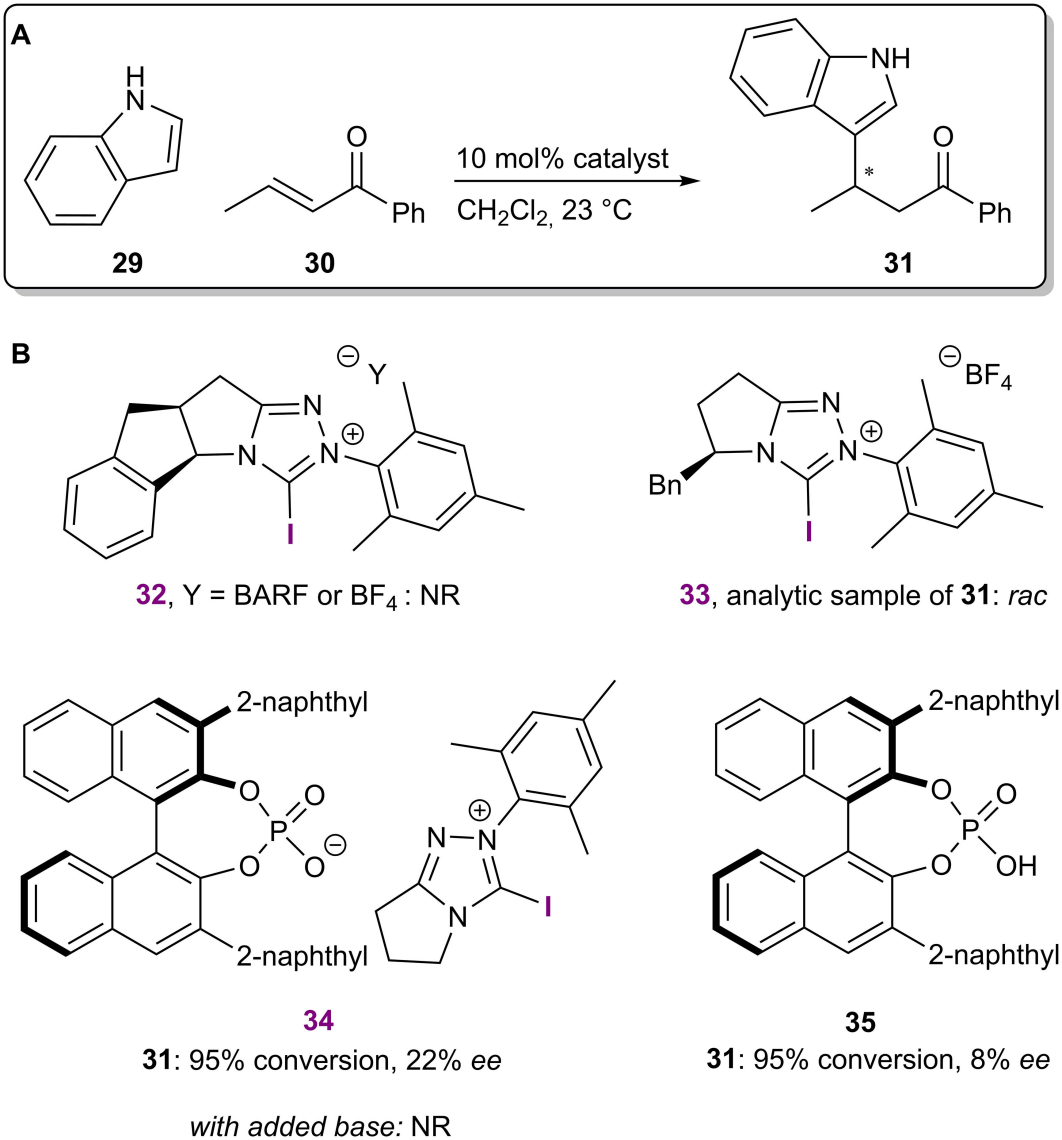
**(A)** Iodo-1,2,4-triazolium salt-catalyzed Michael reaction; **(B)** chiral catalytic systems used in the reaction.

In the same year, 1,2,4-triazolium salts were used to catalyze a Michael reaction between indole **29** and *trans*-crotonophenone **30** (Figure 10A; Squitieri et al., 2019). The use of enantiopure catalysts with chiral cationic backbones did not result in the formation of enantioenriched product **31** (catalysts **32** and **33**; Figure 10B). On the other hand, a low level of asymmetric induction was achieved using salt **34** with an achiral cationic backbone and a chiral BINOL-based phosphate as the counterion. Similar to the previous example, the BINOL-based phosphoric acid **35** yielded the product **31** in a somewhat lower selectivity. The activation of the carbonyl group of **30** was envisioned to come from the XB and ^13^C NMR experiments revealed that the 1,2,4-triazolium salts can interact with some Lewis bases, including carbonyl compounds. Both the selectivity and reactivity seemed to be affected by the presence of the iodoazolium part of the catalyst **34**. However, control experiments carried out with the corresponding phosphoric acid **35** and in the presence of proton scavengers did not support an activation mode by XBs. It was concluded that the true catalytic activity was the result of hidden Brønsted acid catalysis. This is a constant danger associated with the development of XB catalysis, as sufficient levels of catalytic activity are often achieved only with the use of charged azolium-type XB donors. In addition, a recent computational publication explored quinone activation by XBs in a Hantzsch ester reduction reaction (He et al., 2014) and demonstrated that a Brønsted acid-catalyzed pathway should be more favorable (Ser et al., 2019). These examples highlight the importance of control experiments in catalyst development to confirm a mode of activation by XBs.

It should be noted that several chiral XB donors have been featured in publications on XB catalysis (Kniep et al., 2012; He et al., 2014; Kaasik et al., 2019). However, the presence of asymmetric induction was not observed or discussed in these publications. Kaasik et al. (2019) used chiral 1,2,3-triazolium salts to activate imines in an aza-Diels-Alder reaction, which provided a racemic product. Similar XB donors had previously been demonstrated to discriminate between the enantiomers of a chiral thiourea (Kaasik et al., 2017). However, the discrimination of enantiomers of a chiral imine and a chiral amine by these donors was not observed (Peterson et al., 2019). Based on these reports, the authors reasoned that additional noncovalent interactions were also needed to facilitate enantiodiscrimination. In addition, Beer et al. demonstrated the possibility of using XBs to discriminate between chiral anions if bidentate XB donors were used (Lim et al., 2016, 2018; Borissov et al., 2017).

**Figure 11:**
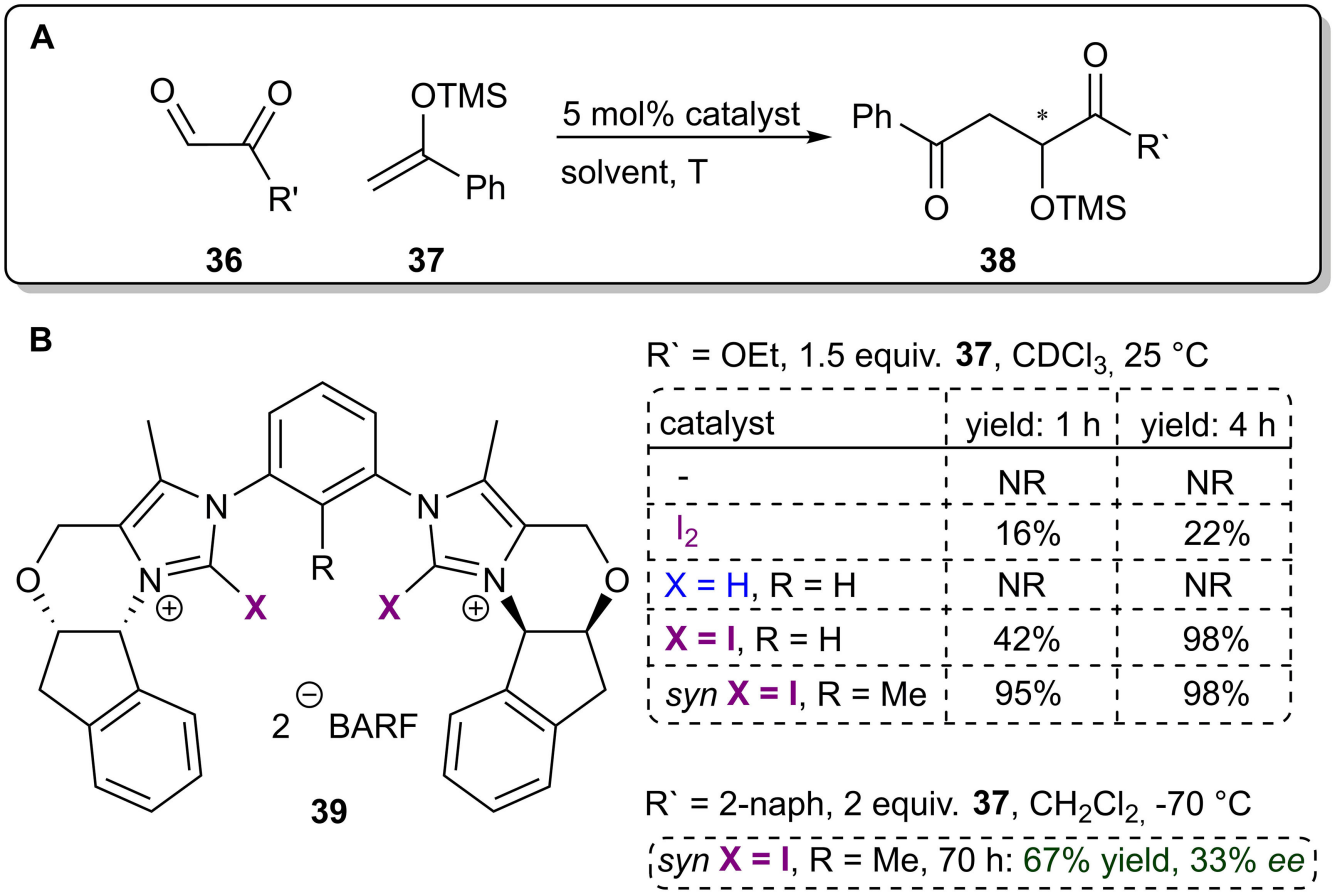
**(A)** Enantioselective XB-catalyzed Mukayama aldol reaction, yields refer to approximate values from Figure 6 in the publication (Sutar et al., 2020); **(B)** influence of the structure of the catalyst and reaction conditions on the reaction outcome.

Recently the Huber group published the first example of a purely XB-based catalyst achieving enantioinduction in a reaction (Figure 11A; Sutar et al., 2020). The novel catalyst **39** featured a bidentate iodoimidazolium core for substrate activation with rigidly fixed “chiral” sidearms to achieve enantioinduction. The performance of the catalyst was improved further by pre-organizing the XB-donating sidearms into the syn configuration by placing a methyl group in the phenyl linker unit. ^1^H NMR experiments confirmed that the chiral XB donor could discriminate between enantiomers of a chiral bidentate diamine. These observations were supported by DFT calculations, which indicated that the differentiation was the result of different spatial orientations of the acceptors toward the XB donor. Therefore, bidentate acceptors **36** featuring adjacent carbonyl groups were used as substrates in a Mukayama aldol reaction with silyl enol ethers **37**. No reaction took place in the absence of the catalyst and in the case of the hydrogen analog of the catalyst (Figure 11B). Furthermore, possible decomposition products of the catalyst were either inactive or less active. These results supported the hypothesis of activation by XBs. The authors proposed that the bidentate nature of the substrate **36** was important both for the activity and the selectivity, as monocarbonyl electrophiles (benzaldehyde and *o*-anisaldehyde) led to products with a decreased selectivity or yield. With the use of the more active prefixed catalyst variant, the enantioselectivity of **38** was increased to 33% *ee* at −70°C. Although quite low in the sense of traditional asymmetric catalysis, this is an important milestone in the development of asymmetric XB catalysis.

**Figure 12:**
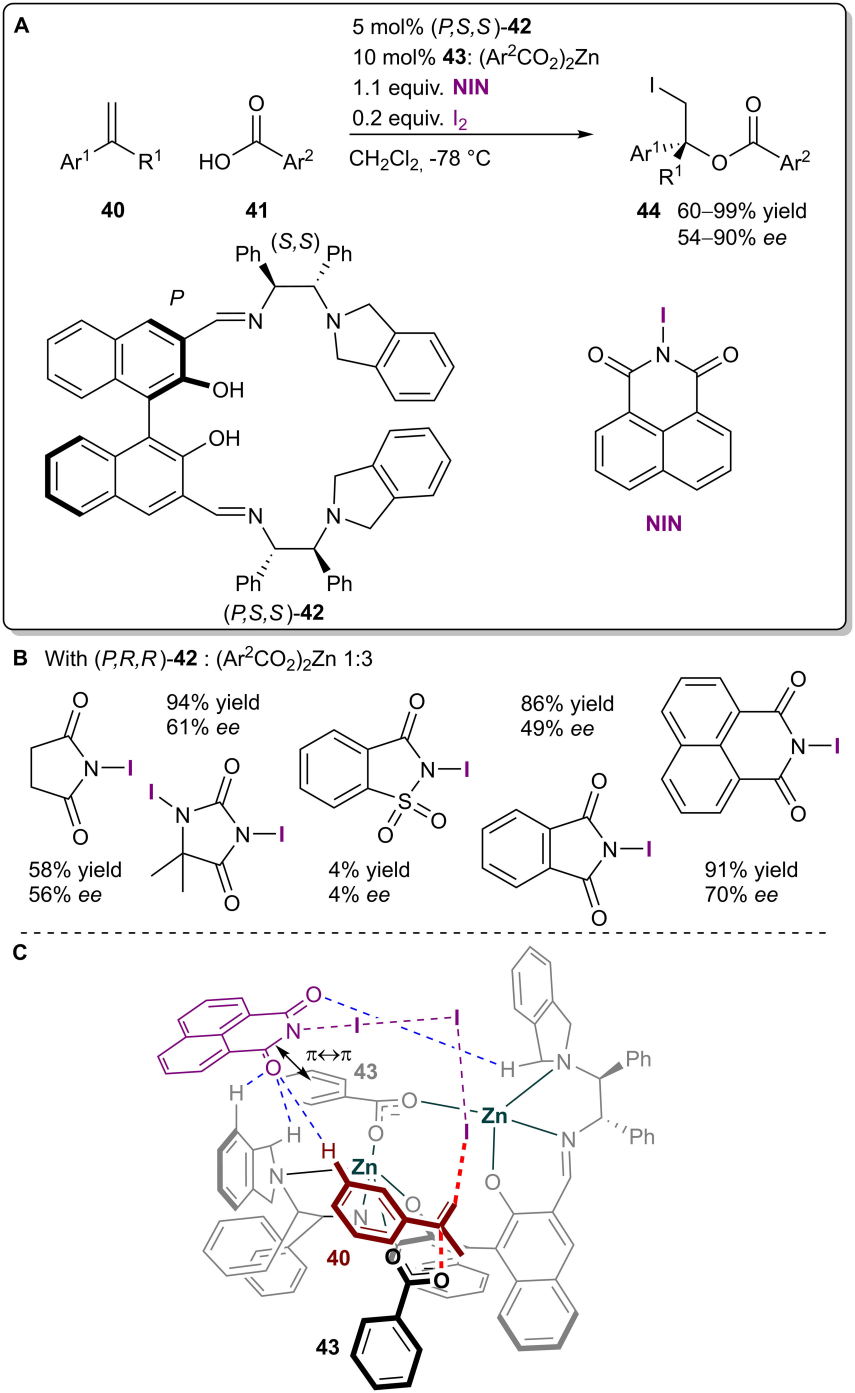
**(A)** Zinc-catalyzed asymmetric iodoesterification utilizing the **I**_**2**_**-NIN** complex as a halogenating agent; **(B)** comparison of the influence of the different halogen sources on the reaction; **(C)** proposed TS structure leading to the major product.

The Arai group has recently published a paper on an enantioselective haloesterfication reaction between styrenes **40** and benzoic acids **41** utilizing a chiral dinuclear zinc complex formed *in situ* from chiral ligand **42** and zinc carboxylate **43** (Figure 12A; Arai et al., 2020). XBs were again used for the generation of active iodinating species. The use of different iodoimides affected both the yield and selectivity of the reaction (Figure 12B). Based on DFT calculations and their earlier study (Arai et al., 2019), the authors proposed a transition state structure, in which a halogen-bonding network activated the styrene **40** from the *Re*-face, while the nucleophilic zinc carboxylate **43** attacked the double bond from the other side, giving the *R*-iodoester (Figure 12C). Hydrogen bonding and π-π interactions were also crucial for the organization of the 3D transition structure. As the exact influence of the different halogenating complexes on the enantioselectivity was not explored, it could be that the π-π stacking ability of the haloimide had a more profound role on stereoselectivity than the XB donor ability of the haloimide. With the optimized conditions at hand, iodoesters **44** were obtained in moderate to excellent yields (60–99%) and moderate to good selectivities (54–90% *ee*).

## XBs in Enhancing Selectivity

**Figure 13:**
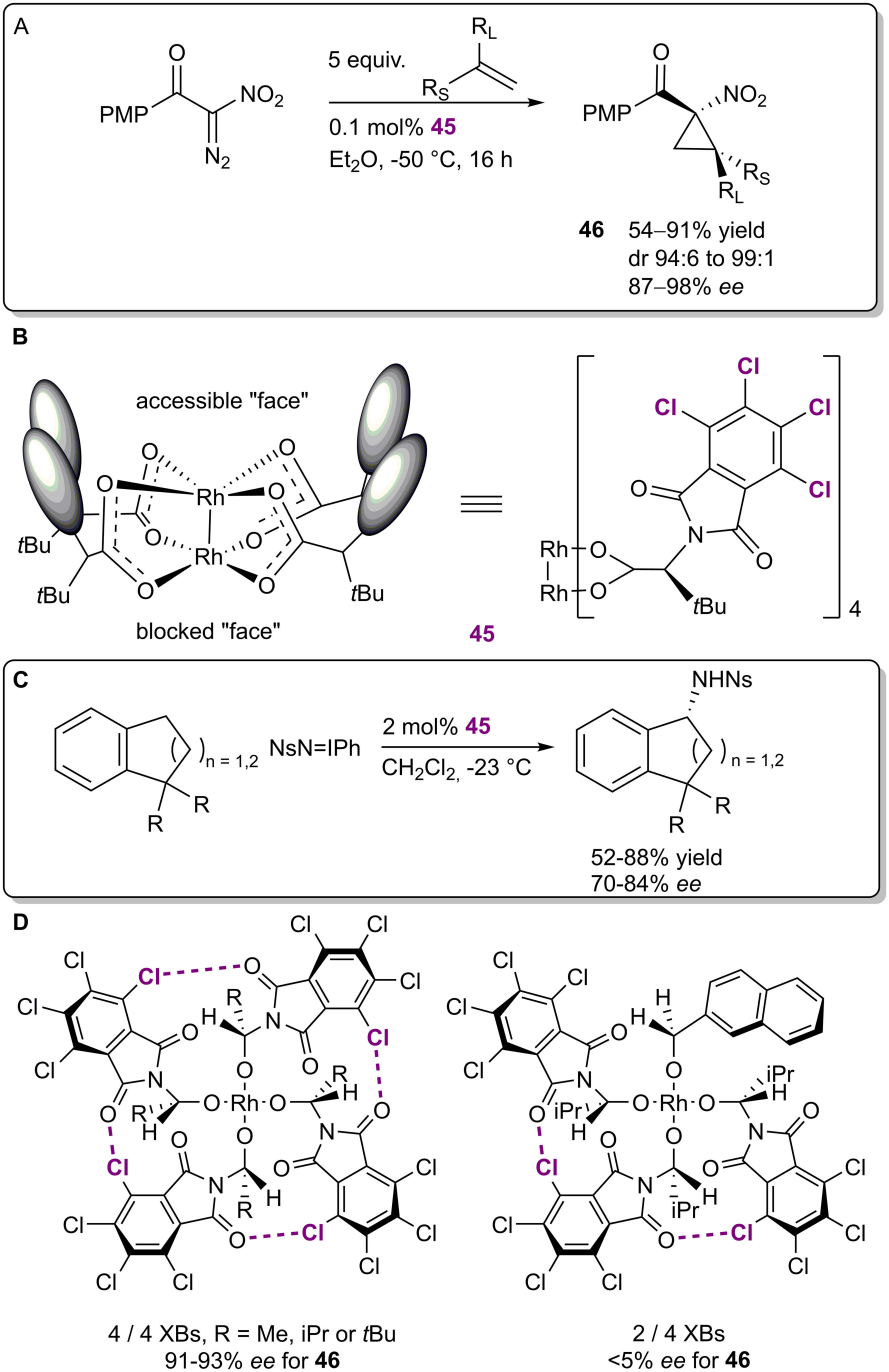
**(A)** Enantioselective Ru-catalyzed cyclopropanation; **(B)** structure of the Ru-catalyst **45**; **(C)** enantioselective Ru-catalyzed amidation; **(D)** top view of two Ru-catalysts depicting the XB networks and their influence on the selectivity of the cyclopropanation.

In 2009, the Charette group published an example of an enantioselective Ru-catalyzed cyclopropanation (Figure 13A; Lindsay et al., 2009) in which halogen bonds were used to fix the ligands of the ruthenium species **45** into a bowl shape (Figure 13B). The cyclopropanes **46** were obtained in moderate to excellent yields (54–91%), excellent diastereoselectivities (94:6–99:1) and good to excellent enantioselectivities (87–98% *ee*). Interestingly, the catalyst **45** was designed and successfully used in an enantioselective amidation reaction by the Hashimoto group as early as 2002 (Figure 13C; Yamawaki et al., 2002). At the time, it was reasoned that the chlorine atoms had a primarily electronic influence on the catalyst, resulting in improved performance compared to other non-halogenated analogs. In contrast, Charette et al. obtained X-ray crystal structures of catalyst **45** and its non-halogenated version, and both had similar bowl-shaped structures. ^1^H-^13^C heteronuclear NOESY NMR experiments revealed that the halogenated version retained its shape in solution, leaving only one ruthenium atom accessible for catalysis, while the non-halogenated analog was more flexible. The difference in flexibility was primarily attributed to the XBs formed among the ligands as observed in crystal structures. In a follow-up report, Charette et al. demonstrated that (by omitting XB aceptor or donor fragments from the catalyst) at least three XBs must form among the ligands to obtain high levels of selectivity, leaving room for catalyst design (Figure 13D; Lindsay and Charette, 2012). In the years since then, catalyst **45** or its analogs have been used in other enantioselective transformations, but catalytic applications have been the focus of the research (for selected examples, see: Goto et al., 2011; Liao et al., 2017; Rodriguez et al., 2018). As the shape of the catalyst and its effect on selectivity have been determined, the importance of the halogen bonding network has not been explored further and often is not mentioned in publications at all.

**Figure 14:**
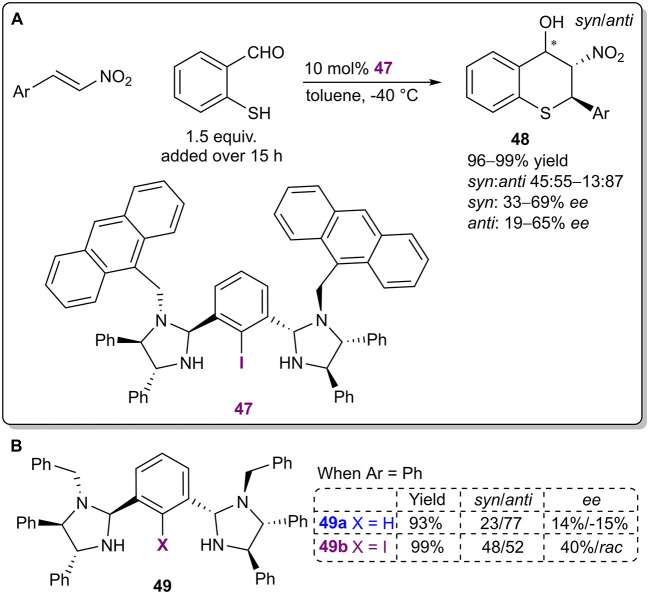
**(A)** Bis(imidazolidine)iodobenzene- catalyzed enantioselective Michael/Henry cascade reaction; **(B)** influence of the halogen atom of the catalyst **49** on the reaction.

The Arai research group developed a chiral bis(imidazolidine)iodobenzene catalyst **47** based on the core of similar NCN-pincer metal complexes (Figure 14A; Arai et al., 2017). The organocatalysts were used in a Michael/Henry cascade reaction to obtain thiochromanes **48** with excellent yields (up to 99%), moderate diastereoselectivities (up to 13:87 *syn*:*anti*) and moderate enantioselectivities (up to 65% *ee* for the major *anti* diastereoisomer). Although the authors have stated that halogen bonding was an important catalyst design element, the exact mode of activation was not discussed, and not enough control experiments were conducted. A difference in stereoselectivity between the iodinated catalyst **49b** and its non-halogenated analog **49a** (40% *ee* and 14% *ee*, respectively, for the minor *syn* diastereomer during initial catalyst screening; Figure 14B) was observed, which can also be attributed to the steric influence of iodine. Notably, it is quite unlikely that an iodophenyl fragment without electron-withdrawing substituents forms a strong enough XB with the formyl group to single-handedly activate it. Due to the slow addition of the aldehyde throughout the reaction, the activity of the catalysts is also difficult to establish. In both cases the product was obtained in excellent yields (99 and 93%, respectively). Further catalyst optimization resulted in an increase in the previously mentioned selectivities with the use of sterically larger substituents in the catalyst instead of the benzyl substituents.

**Figure 15:**
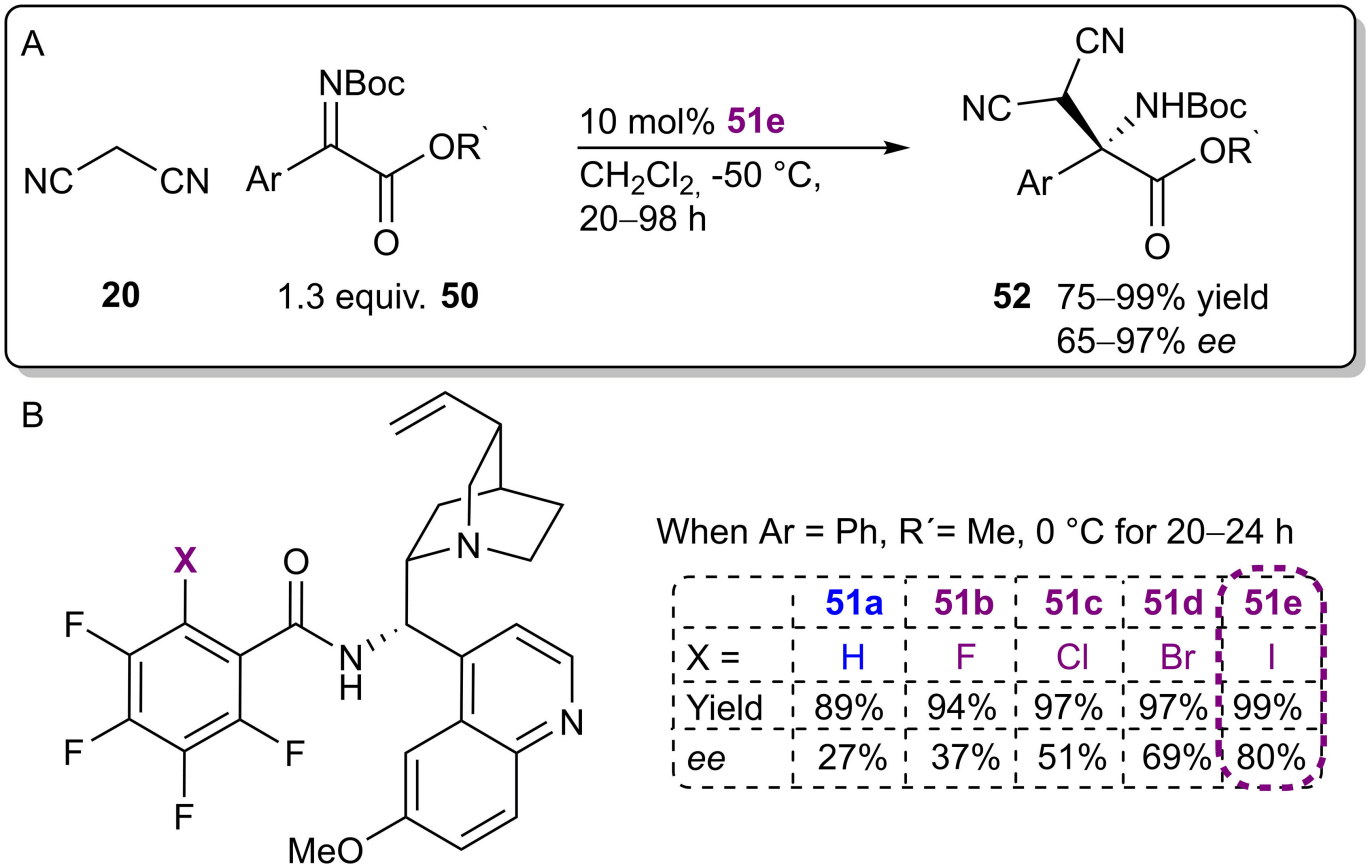
**(A)** Enantioselective Mannich reaction catalyzed by bifunctional Brønsted base/XB catalyst **51e**; **(B)** influence of the halogen atom of the catalyst **51** on the reaction.

The Arai group continued research on their bifunctional Brønsted base/XB catalyst and recently published an example in which the catalysts were used to activate malononitrile **20** and ketiminoesters **50** in a Mannich reaction (Figure 15A; Kuwano et al., 2020). Catalyst optimization studies revealed a significant dependence between the donor atom and reaction selectivity that corresponded to the polarizability order of the halogens (82%>69%>51%>37%>27% *ee* for I>Br>Cl>F>H; Figure 15B). However, in contrast to their first study utilizing this catalytic system, the activity of the catalyst **51** was not greatly affected by this change (yields ranged from 89 to 99%). ^19^F NMR titration experiments also supported the participation of XBs. A truncated analog of catalyst **51e** without a chiral alkaloid fragment interacted more strongly with the imine than did the non-halogenated analog (affinity constant with values of K_a_ 0.90 M^−1^ and K_a_ 0.17 M^−1^, respectively). Based on these observation, a catalytic cycle similar to the one described in their earlier publication was proposed: the electrophilic imine was activated by an XB to its carbonyl group (see Figure 8C). In future studies, it would be of interest to ascertain the role of the acidic amid *N*H proton. All in all, under optimized conditions enantioenriched products **52** were obtained in good to excellent yields (75–99%) and moderate to excellent selectivities (65–97% *ee*) using catalyst **51e**.

## Conclusion and Perspective

About half of the examples, that received an in dept examination, have been published in the past 2 years, which demonstrates a significant increase in the interest in asymmetric XB catalysis. As mentioned throughout the review, the participation of XBs has not been acknowledged in several instances or in initial studies. This leads to the realization that there could be other examples in the literature in which the crucial role of XBs has remained unnoticed. Nevertheless, both the acknowledgment that XBs could participate in catalytic enantioselective processes and the deliberate use of XBs in these processes has increased.

So far, the use of cationic XB donors to achieve asymmetric induction has not been very successful, leading to at best moderate stereoselectivities. One would have to concur with the statement by Huber (Sutar and Huber, 2019) that the combination of the size of the halogen substituent, the length of its covalent bond and the linearity requirements of the corresponding XB make the task of achieving asymmetric induction very difficult. In principle, the relevance of XBs in purely XB-based catalysts should be simpler to confirm than the relevance of XBs in bifunctional catalysts. However, the possibility of other competing pathways in the case of charge-assisted XB catalysis hinders its development further. In contrast, bifunctional systems that have not utilized XBs as the primary mode of activation, but as secondary elements (that is, as organizing units or as secondary activating units) have led to better results. More surprisingly, the weaker neutral XB donor motifs are prevalent in these examples compared to charge-assisted XB donor motifs. With the publication of the first example utilizing only XBs to achieve asymmetric induction, the field might have reached a turning point in its development with new purely XB catalytic examples hopefully following in the near future.

It is also noteworthy that XB-donating units have been used in, or in combination with, very different catalyst types (Lewis bases, PTCs, Brønsted bases, and metal catalysts), demonstrating the wide reach of XBs. In addition, the examples discussed in this review cover a wide range of different reactions. Therefore, XBs have a lot of potential for applications in asymmetric catalysis. However, as the relevant information is scattered among a diverse set of examples it will not be a trivial task to use this knowledge successfully and this will depend on the ingenuity of the scientist. Acknowledging the influence of XBs on the reactivity or the selectivity of the reaction after the fact is much simpler than predicting this influence beforehand and then applying it successfully.

## Author Contributions

Both authors contributed substantially to the work reported, have read, and agreed to the published version of the manuscript.

## Conflict of Interest

The authors declare that the research was conducted in the absence of any commercial or financial relationships that could be construed as a potential conflict of interest. The handling editor declared a shared affiliation, though no other collaboration, with the authors MK and TK.

## References

[B1] Arai T. Horigane K. Suzuki T. K. Itoh R. Yamanaka M. (2020). Catalytic asymmetric iodoesterification of simple alkenes. Angew. Chem. Int. Ed. 59, 12680–12683. 10.1002/anie.202003886 32342634

[B2] Arai T. Horigane K. Watanabe O. Kakino J. Sugiyama N. Makino H.. (2019). Association of halogen bonding and hydrogen bonding in metal acetate-catalyzed asymmetric halolactonization. iScience 12, 280–292. 10.1016/j.isci.2019.01.029 30731356 PMC6365408

[B3] Arai T. Kajikawa S. Matsumura E. (2013). The role of ni-carboxylate during catalytic asymmetric iodolactonization using pybidine-ni(OAc). Synlett 24, 2045–2048. 10.1055/s-0033-1339676

[B4] Arai T. Kojima T. Watanabe O. Itoh T. Kanoh H. (2015a). Recyclable Poly-Zn_3_(OAc)_4_-3,3′-Bis(aminoimino)binaphthoxide catalyst for asymmetric iodolactonization. ChemCatChem 7, 3234–3238. 10.1002/cctc.201500842

[B5] Arai T. Suzuki T. Inoue T. Kuwano S. (2017). Chiral Bis(imidazolidine)iodobenzene (I-Bidine) organocatalyst for thiochromane synthesis using an asymmetric michael/henry reaction. Synlett 28, 122–127. 10.1055/s-0036-1588614

[B6] Arai T. Watanabe O. Yabe S. Yamanaka M. (2015b). Catalytic asymmetric iodocyclization of *N-* tosyl alkenamides using aminoiminophenoxy copper carboxylate: a concise synthesis of chiral 8-Oxa-6-azabicyclo[3.2.1]octanes. Angew. Chem. Int. Ed. 54, 12767–12771. 10.1002/anie.201505748 26364981

[B7] Bamberger J. Ostler F. Mancheño O. G. (2019). Frontiers in halogen and chalcogen-bond donor organocatalysis. ChemCatChem 11, 5198–5211. 10.1002/cctc.201901215 31894187 PMC6919929

[B8] Beale T. M. Chudzinski M. G. Sarwar M. G. Taylor M. S. (2013). Halogen bonding in solution: thermodynamics and applications. Chem. Soc. Rev. 42, 1667–1680. 10.1039/C2CS35213C 22858664

[B9] Borissov A. Lim J. Y. C. Brown A. Christensen K. E. Thompson A. L. Smith M. D.. (2017). Neutral iodotriazole foldamers as tetradentate halogen bonding anion receptors. Chem. Commun. 53, 2483–2486. 10.1039/C7CC00727B 28181604

[B10] Breugst M. Koenig J. J. (2020). σ-Hole interactions in catalysis. Eur. J. Org. Chem. 2020, 5473–5487. 10.1002/ejoc.202000660

[B11] Brinck T. Murray J. S. Politzer P. (1992). Surface electrostatic potentials of halogenated methanes as indicators of directional intermolecular interactions. Int. J. Quantum Chem. 44, 57–64. 10.1002/qua.560440709

[B12] Brown R. S. (1997). Investigation of the early steps in electrophilic bromination through the study of the reaction with sterically encumbered olefins. Acc. Chem. Res. 30, 131–137. 10.1021/ar960088e

[B13] Bruckmann A. Pena M. A. Bolm C. (2008). Organocatalysis through halogen-bond activation. Synlett 2008, 900–902. 10.1055/s-2008-1042935

[B14] Cai Y. Liu X. Zhou P. Feng X. (2019). asymmetric catalytic halofunctionalization of α,β-unsaturated carbonyl compounds. J. Org. Chem. 84, 1–13. 10.1021/acs.joc.8b01951 30339377

[B15] Cavallo G. Metrangolo P. Milani R. Pilati T. Priimagi A. Resnati G.. (2016). The halogen bond. Chem. Rev. 116, 2478–2601. 10.1021/acs.chemrev.5b00484 26812185 PMC4768247

[B16] Chan Y. C. Yeung Y. Y. (2019). Halogen-bond-catalyzed addition of carbon-based nucleophiles to n-acylimminium ions. Org. Lett. 21, 5665–5669. 10.1021/acs.orglett.9b02006 31282678

[B17] Cheng Y. A. Yu W. Z. Yeung Y. Y. (2014). Recent advances in asymmetric intra- and intermolecular halofunctionalizations of alkenes. Org. Biomol. Chem. 12, 2333–2343. 10.1039/c3ob42335b 24595745

[B18] Christopherson J. C. Topić F. Barrett C. J. Friščić T. (2018). Halogen-bonded cocrystals as optical materials: next-generation control over light-matter interactions. Cryst. Growth Des. 18, 1245–1259. 10.1021/acs.cgd.7b01445

[B19] Claraz A. Masson G. (2018). Asymmetric iodine catalysis-mediated enantioselective oxidative transformations. Org. Biomol. Chem. 16, 5386–5402. 10.1039/C8OB01378K 30024581

[B20] Clark T. Hennemann M. Murray J. S. Politzer P. (2007). “Halogen bonding: the σ-hole,” in *Proceedings of “Modeling interactions in biomolecules II”*, Prague, September 5–9th, 2005. J. Mol. Model. 13, 291–296. 10.1007/s00894-006-0130-2 16927107

[B21] Colin J. J. de Claubry H. (1814). Sur le combinaisons de l'iode avec les substances végétales et animales. Ann. Chim 90, 87–100.

[B22] Cresswell A. J. Eey S. T. C. Denmark S. E. (2015). Catalytic, Stereoselective dihalogenation of alkenes: challenges and opportunities. Angew. Chem., Int. Ed. 54, 15642–15682. 10.1002/anie.201507152 26630449 PMC5072131

[B23] Desiraju G. R. Shing Ho P. Kloo L. Legon A. C. Marquardt R. Metrangolo P. (2013). Definition of the halogen bond (IUPAC recommendations 2013). Pure Appl. Chem. 85, 1711–1713. 10.1351/PAC-REC-12-05-10

[B24] Flores A. Cots E. Bergès J. Muñiz K. (2019). Enantioselective Iodine(I/III) catalysis in organic synthesis. Adv. Synth. Catal. 361, 2–25. 10.1002/adsc.201800521

[B25] Gilday L. C. Robinson S. W. Barendt T. A. Langton M. J. Mullaney B. R. Beer P. D. (2015). Halogen bonding in supramolecular chemistry. Chem. Rev. 115, 7118–7195. 10.1021/cr500674c 26165273

[B26] Goto T. Takeda K. Shimada N. Nambu H. Anada M. Shiro M.. (2011). Highly enantioselective cyclopropenation reaction of 1-alkynes with α-alkyl-α-diazoesters catalyzed by dirhodium(II) carboxylates. Angew. Chem. Int. Ed. 50, 6803–6808. 10.1002/anie.201101905 21674740

[B27] He W. Ge Y. C. Tan C. H. (2014). Halogen-bonding-induced hydrogen transfer to C=N bond with hantzsch ester. Org. Lett. 16, 3244–3247. 10.1021/ol501259q 24904974

[B28] Heinen F. Engelage E. Cramer C. J. Huber S. M. (2020). Hypervalent Iodine(III) compounds as biaxial halogen bond *donors*. J. Am. Chem. Soc. 142, 8633–8640. 10.1021/jacs.9b13309 32286829 PMC7252947

[B29] Iwase S. Suzuki Y. Tanaka S. Kitamura M. (2020). CpRu/Brønsted acid-catalyzed enantioselective dehydrative cyclization of pyrroles n-tethered with allylic alcohols. Org. Lett. 22, 1929–1933. 10.1021/acs.orglett.0c00290 32069061

[B30] Jentzsch A. V. (2015). Applications of halogen bonding in solution. Pure Appl. Chem. 87, 15–41. 10.1515/pac-2014-0807

[B31] Kaasik M. Kaabel S. Kriis K. Järving I. Aav R. Rissanen K.. (2017). Synthesis and characterisation of chiral triazole-based halogen-bond donors: halogen bonds in the solid state and in solution. Chem. Eur. J. 23, 7337–7344. 10.1002/chem.201700618 28266794

[B32] Kaasik M. Metsala A. Kaabel S. Kriis K. Järving I. Kanger T. (2019). Halo-1,2,3-triazolium salts as halogen bond donors for the activation of imines in dihydropyridinone synthesis. J. Org. Chem. 84, 4295–4303. 10.1021/acs.joc.9b00248 30855960

[B33] Kniep F. Jungbauer S. H. Zhang Q. Walter S. M. Schindler S. Schnapperelle I.. (2013). Organocatalysis by neutral multidentate halogen-bond donors. Angew. Chem. Int. Ed. 52, 7028–7032. 10.1002/anie.201301351 23649719

[B34] Kniep F. Rout L. Walter S. M. Bensch H. K. V. Jungbauer S. H. Herdtweck E.. (2012). 5-Iodo-1,2,3-triazolium-based multidentate halogen-bond donors as activating reagents. Chem. Commun. 48, 9299–9301. 10.1039/c2cc34392d 22875079

[B35] Kristianslund R. Tungen J. E. Hansen T. V. (2019). Catalytic enantioselective iodolactonization reactions. Org. Biomol. Chem. 17, 3079–3092. 10.1039/C8OB03160F 30806424

[B36] Kuwano S. Nishida Y. Suzuki T. Arai T. (2020). Catalytic asymmetric mannich-type reaction of malononitrile with N-Boc α-ketiminoesters using chiral organic base catalyst with halogen bond donor functionality. Adv. Synth. Catal. 362, 1674–1678. 10.1002/adsc.202000092

[B37] Kuwano S. Suzuki T. Hosaka Y. Arai T. (2018). A chiral organic base catalyst with halogen-bonding-donor functionality: asymmetric Mannich reactions of malononitrile with: N -Boc aldimines and ketimines. Chem. Commun. 54, 3847–3850. 10.1039/C8CC00865E 29594299

[B38] Kwon H. Y. Park C. M. Lee S. B. Youn J. H. Kang S. H. (2008). Asymmetric iodocyclization catalyzed by salen-CrIIICl: its synthetic application to swainsonine. Chem. Eur. J. 14, 1023–1028. 10.1002/chem.200701199 17972261

[B39] Lenoir D. Chiappe C. (2003). What is the nature of the first-formed intermediates in the electrophilic halogenation of alkenes, alkynes, and allenes? Chem. Eur. J. 9, 1036–1044. 10.1002/chem.200390097 12596140

[B40] Li B. Zang S.-Q. Wang L.-Y. Mak T. C. W. (2016). Halogen bonding: a powerful, emerging tool for constructing high-dimensional metal-containing supramolecular networks. Coord. Chem. Rev. 308, 1–21. 10.1016/j.ccr.2015.09.005

[B41] Liao K. Pickel T. C. Boyarskikh V. Bacsa J. Musaev D. G. Davies H. M. L. (2017). Site-selective and stereoselective functionalization of non-activated tertiary C-H bonds. Nature 551, 609–613. 10.1038/nature24641 29156454

[B42] Lim J. Y. C. Marques I. Félix V. Beer P. D. (2018). Chiral halogen and chalcogen bonding receptors for discrimination of stereo- and geometric dicarboxylate isomers in aqueous media. Chem. Commun. 54, 10851–10854. 10.1039/C8CC06400H 30199082

[B43] Lim J. Y. C. Marques I. Ferreira L. Félix V. Beer P. D. (2016). Enhancing the enantioselective recognition and sensing of chiral anions by halogen bonding. Chem. Commun. 52, 5527–5530. 10.1039/C6CC01701K 27021913

[B44] Lindsay V. N. G. Charette A. B. (2012). Design and synthesis of chiral heteroleptic rhodium(II) carboxylate catalysts: experimental investigation of halogen bond rigidification effects in asymmetric cyclopropanation. ACS Catal. 2, 1221–1225. 10.1021/cs300214v

[B45] Lindsay V. N. G. Lin W. Charette A. B. (2009). Experimental evidence for the all-up reactive conformation of chiral rhodium(II) carboxylate catalysts: enantioselective synthesis of cis-cyclopropane α-amino acids. J. Am. Chem. Soc. 131, 16383–16385. 10.1021/ja9044955 19860407

[B46] Lu Y. Nakatsuji H. Okumura Y. Yao L. Ishihara K. (2018). Enantioselective halo-oxy- and halo-azacyclizations induced by chiral amidophosphate catalysts and halo-lewis acids. J. Am. Chem. Soc. 140, 6039–6043. 10.1021/jacs.8b02607 29708750

[B47] Mahlau M. List B. (2013). Asymmetric counteranion-directed catalysis: Concept, definition, and applications. Angew. Chem., Int. Ed. 52, 518–533. 10.1002/anie.201205343 23280677

[B48] Metrangolo P. Neukirch H. Pilati T. Resnati G. (2005). Halogen bonding based recognition processes: a world parallel to hydrogen bonding. Acc. Chem. Res. 38, 386–395. 10.1021/ar0400995 15895976

[B49] Mizar P. Burrelli A. Günther E. Söftje M. Farooq U. Wirth T. (2014). Organocatalytic stereoselective iodoamination of alkenes. Chem. Eur. J. 20, 13113–13116. 10.1002/chem.201404762 25156303

[B50] Montaña Á. M. (2017). The σ and π Holes. The halogen and tetrel bondings: their nature, importance and chemical, biological and medicinal implications. ChemistrySelect 2, 9094–9112. 10.1002/slct.201701676

[B51] Murai K. Fujioka H. (2013). Recent progress in organocatalytic asymmetric halocyclization. Heterocycles 87, 763–805. 10.3987/REV-12-762

[B52] Nakatsuji H. Sawamura Y. Sakakura A. Ishihara K. (2014). Cooperative activation with chiral nucleophilic catalysts and n-haloimides: enantioselective iodolactonization of 4-arylmethyl-4-pentenoic acids. Angew. Chem. Int. Ed. 53, 6974–6977. 10.1002/anie.201400946 24840957

[B53] Nepal B. Scheiner S. (2015). Substituent effects on the binding of halides by neutral and dicationic bis(triazolium) receptors. J. Phys. Chem. A 119, 13064–13073. 10.1021/acs.jpca.5b09738 26645536

[B54] Parra A. (2019). Chiral hypervalent iodines: active players in asymmetric synthesis. Chem. Rev. 119, 12033–12088. 10.1021/acs.chemrev.9b00338 31741377

[B55] Pearson R. G. (1963). Hard and soft acids and bases. J. Am. Chem. Soc. 85, 3533–3539. 10.1021/ja00905a001

[B56] Peterson A. Kaasik M. Metsala A. Järving I. Adamson J. Kanger T. (2019). Tunable chiral triazole-based halogen bond donors: assessment of donor strength in solution with nitrogen-containing acceptors. RSC Adv. 9, 11718–11721. 10.1039/C9RA01692A PMC9063393 35517004

[B57] Rissanen K. (2008). Halogen bonded supramolecular complexes and networks. CrystEngComm 10, 1107–1113. 10.1039/b803329n

[B58] Robertson C. C. Perutz R. N. Brammer L. Hunter C. A. (2014). A solvent-resistant halogen bond. Chem. Sci. 5, 4179–4183. 10.1039/C4SC01746C

[B59] Robertson C. C. Wright J. S. Carrington E. J. Perutz R. N. Hunter C. A. Brammer L. (2017). Hydrogen bonding: vs. halogen bonding: the solvent decides. Chem. Sci. 8, 5392–5398. 10.1039/C7SC01801K 28970918 PMC5585772

[B60] Rodriguez K. X. Pilato T. C. Ashfeld B. L. (2018). An unusual stereoretentive 1,3-quaternary carbon shift resulting in an enantioselective RhII-catalyzed formal [4+1]-cycloaddition between diazo compounds and vinyl ketenes. Chem. Sci. 9, 3221–3226. 10.1039/C8SC00020D 29844895 PMC5931190

[B61] Seki T. Tanaka S. Kitamura M. (2012). Enantioselective synthesis of pyrrolidine-, Piperidine-, and azepane-type N -heterocycles with α-alkenyl substitution: the CpRu-catalyzed dehydrative intramolecular N -allylation approach. Org. Lett. 14, 608–611. 10.1021/ol203218d 22196102

[B62] Ser C. T. Yang H. Wong M. W. (2019). Iodoimidazolinium-catalyzed reduction of quinoline by hantzsch ester: halogen bond or brønsted acid catalysis. J. Org. Chem. 84, 10338–10348. 10.1021/acs.joc.9b01494 31283228

[B63] Squitieri R. A. Fitzpatrick K. P. Jaworski A. A. Scheidt K. A. (2019). Synthesis and evaluation of azolium-based halogen-bond donors. Chem- Eur. J. 60208, 10069–10073. 10.1002/chem.201902298 31112630

[B64] Sutar R. Huber S. M. (2019). Catalysis of organic reactions through halogen bonding. ACS Catal. 9, 9622–9639. 10.1021/acscatal.9b02894

[B65] Sutar R. L. Engelage E. Stoll R. Huber S. M. (2020). Bidentate chiral bis(imidazolium)-based halogen-bond donors: synthesis and applications in enantioselective recognition and catalysis. Angew. Chem. Int. Ed. 59, 6806–6810. 10.1002/anie.201915931 32045504 PMC7187470

[B66] Suzuki Y. Seki T. Tanaka S. Kitamura M. (2015). Intramolecular tsujitrost-type allylation of carboxylic acids: asymmetric synthesis of highly π-allyl donative lactones. J. Am. Chem. Soc. 137, 9539–9542. 10.1021/jacs.5b05786 26199057

[B67] Tanaka S. Seki T. Kitamura M. (2009). Asymmetrie dehydrative cyclization of ω-hydroxy allyl alcohols catalyzed by ruthenium complexes. Angew. Chem. Int. Ed. 48, 8948–8951. 10.1002/anie.200904671 19842154

[B68] Teng B. Chen W. Dong S. Kee C. W. Gandamana D. A. Zong L.. (2016). Pentanidium- and bisguanidinium-catalyzed enantioselective alkylations using silylamide as brønsted probase. J. Am. Chem. Soc. 138, 9935–9940. 10.1021/jacs.6b05053 27447024

[B69] Tepper R. Schubert U. S. (2018). Halogen bonding in solution: anion recognition, templated self-assembly, and organocatalysis. Angew. Chem. Int. Ed. 57, 6004–6016. 10.1002/anie.201707986 29341377

[B70] Troff R. W. Mäkelä T. Topic F. Valkonen A. Raatikainen K. Rissanen K. (2013). Alternative motifs for halogen bonding. Eur. J. Org. Chem. 2013, 1617–1637. 10.1002/ejoc.201201512

[B71] Tungen J. E. Nolsoe J. M. J. Hansen T. V. (2012). Asymmetric iodolactonization utilizing chiral squaramides. Org. Lett. 14, 5884–5887. 10.1021/ol302798g 23148494

[B72] Turunen L. Erdélyi M. (2020). Halogen bonds of halonium ions. Chem. Soc. Rev. 49, 2688–2700. 10.1039/D0CS00034E 32211673

[B73] Veitch G. E. Jacobsen E. N. (2010). Tertiary aminourea-catalyzed enantioselective iodolactonization. Angew. Chem. Int. Ed. 49, 7332–7335. 10.1002/anie.201003681 20803601 PMC2993562

[B74] Wang H. Wang W. Jin W. J. (2016). σ-hole bond vs π-hole bond: a comparison based on halogen bond. Chem. Rev. 116, 5072–5104. 10.1021/acs.chemrev.5b00527 26886515

[B75] Yamawaki M. Tsutsui H. Kitagaki S. Anada M. Hashimoto S. (2002). Dirhodium(II) tetrakis[N-tetrachlorophthaloyl-(S)-tert-leucinate]: a new chiral Rh(II) catalyst for enantioselective amidation of C-H bonds. Tetrahedron Lett. 43, 9561–9564. 10.1016/S0040-4039(02)02432-2

[B76] Yang H. Wong M. W. (2020). Application of halogen bonding to organocatalysis: a theoretical perspective. Molecules 25:1045. 10.3390/molecules25051045 32110944 PMC7179134

[B77] Zhang Y. Han J. Liu Z. J. (2015). Diaryliodonium salts as efficient Lewis acid catalysts for direct three component mannich reactions. RSC Adv. 5, 25485–25488. 10.1039/C5RA00209E

[B78] Zong L. Ban X. Kee C. W. Tan C. H. (2014). Catalytic enantioselective alkylation of sulfenate anions to chiral heterocyclic sulfoxides using halogenated pentanidium salts. Angew. Chem. Int. Ed. 53, 11849–11853. 10.1002/anie.201407512 25209332

[B79] Zong L. Du S. Chin K. F. Wang C. Tan C. H. (2015). Enantioselective synthesis of quaternary carbon stereocenters: addition of 3-substituted oxindoles to vinyl sulfone catalyzed by pentanidiums. Angew. Chem. Int. Ed. 54, 9390–9393. 10.1002/anie.201503844 26179829

